# PGM1 and ENO1 Promote the Malignant Progression of Bladder Cancer via Comprehensive Analysis of the m6A Signature and Tumor Immune Infiltration

**DOI:** 10.1155/2022/8581805

**Published:** 2022-02-24

**Authors:** Jinglin Zhao, Shu Huang, Dingji Tan, Kaiqing Yang, Mingyue Chen, Xiongfei Jia, Xiaoqin Mao

**Affiliations:** ^1^Institute of Laboratory Medicine, The Affiliated Hospital of Kunming University of Science and Technology, Kunming, China; ^2^Institute of Laboratory Medicine, The First People's Hospital of Yunnan Province, Kunming 650000, Yunnan, China; ^3^Kunming Medical University, Faculty of Medical Laboratory Science, The Third Affiliated Hospital of Kunming University, Kunming 650000, Yunnan, China; ^4^Department of Neurology, Affiliated Hospital of North Sichuan Medical College, Institute of Neurological Diseases, North Sichuan Medical College, Nanchong 637000, Sichuan, China; ^5^Department of Clinical Laboratory, 920th Hospital of Joint Logistics Support Force of Chinese People's Liberation Army, Kunming 650000, Yunnan, China

## Abstract

**Background:**

While N^6^-methyladenosine (m6A) modification of RNA and the tumor immune microenvironment both influence the progression of cancer, little attention has been paid to interactions between these two factors. Thus, we systematically explored potential biomarkers in the malignant progression of bladder urothelial carcinoma (BLCA) via combining expression of m6A methylation regulators with tumor immune infiltration.

**Methods:**

We extracted m6A regulators from published literature, downloaded BLCA RNA-seq and clinical information from the Cancer Genome Atlas database, and integrated three main bioinformatic methods and qPCR to explore the biological variations in the malignant progression of BLCA.

**Results:**

FTO, IGF2BP3, and YTHDC1 have a significant difference in bladder cancer and prognosis. Two subgroups (clusters 1 and 2) were identified according to three key m6A regulators; cluster 1 was preferentially associated with poor prognosis and immune infiltration relative to cluster 2 significantly. We further identified PGM1 and ENO1 as potential prognostic biomarkers, as they were correlated with FTO and IGF2BP3 positively but with YTHDC1, negatively. M2 macrophage and TFH cells were highly infiltrated in BLCA and were associated with BLCA prognosis. Finally, PGM1 and ENO1 were correlated with M2 macrophage and TFH cells and their surface markers CD163and CXCR5.

**Conclusions:**

PGM1 and ENO1 are highly correlated with the malignant progression of BLCA, and the expression of these genes may be new indicators for the diagnosis and prognosis of BLCA.

## 1. Introduction

Bladder urothelial carcinoma (BLCA) is a type of therioma that has high rates of morbidity and mortality. BLCA typically occurs in the bladder mucosa, and it is the most commonly diagnosed therioma in the genitourinary system [1, 2]. Approximately 75 000 newly diagnosed cases of BLCA were in the United States in 2015 [3], of which approximately 16 000 died of this malignant tumor. Adding to the seriousness of this disease, the 3-year survival rate is reduced from 50% to 25% if the tumor is invasive [4–6]. Therefore, finding new therapeutic targets is important so that the diagnosis and treatment of BLCA can be improved.

The growing interest in immunotherapy has led to the discovery that immune infiltration in the tumor microenvironment (TME) plays an essential action in the occurrence and development of tumors and affects clinical prognoses [7, 8]. Some of the genomic alterations that characterize cancer cause the production of tumor antigens, which are recognized by the immune system as non-autogenic sources, and trigger cellular immune responses. Studies have shown that cells of the adaptive and innate immune systems infiltrate into the TME and regulate tumor progression [9]. For example, Yi et al. [10] found that the imbalance of the immune system plays a crucial role in the progress of head and neck squamous cell carcinomas and that many cytokines and immunosuppressive cells in the microenvironment of these tumors facilitate immune escape. Therefore, understanding immune infiltration in the TME is likely significant to improving response rates and developing new immunotherapy strategies.

With the continuous development of high-throughput sequencing techniques, the modification of RNA by methylation has increasing attention [11, 12]. Since the presence of the m6A modification of mRNA was first detected in the 1970s by Rottman et al., and an N^6^-methyladenosine transferase (METTL3) was first identified by Bokar et al. in 1997, studies of the m6A modification have become increasingly mature [13–15].

The m6A modification mainly occurs on adenine in a consensus sequence known as “RRACH,” and its function is mainly determined by enzymes known as “Writers,” “Erasers,” and “Readers” [16–18]. The m6A modification has been shown to play a crucial role in gene expression regulation, and alterations of its regulatory mechanisms have been related to various human diseases, including cancers [19–21]. For example, METTL14, which is a component of a writer enzyme complex, inhibits the metastasis of liver cancer by altering the modification of miRNA and affecting the generation and processing of miR-126 [22, 23]. In addition, the increased expression of ALKB H5 RNA demethylase regulates m6A modifications within the nucleus and has been found to negatively correlate with the prognosis of patients with glioblastoma [23].

The significance of the m6A modification has been reported for (in) many cancer types, such as gastric, renal, and lung cancer [24–27]; however, the role of occurrence in the malignant development of BLCA and its prognostic significance are still unclear. Particularly interesting in this regard is the potential function of m6A methylation regulators in the BLCA tumor immune microenvironment (TIME). The reports related to tumor immune interactions have revealed that the regulators of m6A are promising targets to enhance the clinical response of immunotherapy [11]. For example, Han et al. [9] found that the degree of CD8^+^ T cells and natural killer cells in YTHDF1-deficient mice was considerably higher than that in wild-type mice and that these immune cells induced an enhanced antitumor response.

In this study, to investigate the potential biomarkers regarding the malignant progression of BLCA, the consensus clustering analyses of the TCGA and GEO databases were conducted to identify candidate genes that may be involved in m6A modification and immune infiltration in BLCA. The analyses of differentially expressed genes and their prognostic value, combined with the characteristics of TIME, were used to identify the potential biomarkers for clinical diagnosis and therapy.

## 2. Materials and Methods

### 2.1. Acquisition and Processing of Raw Data

All data were obtained from the TCGA data portal (https://portal.gdc.cancer.gov/) using the Genomic Data Commons Data Transfer Tool. The cohort consists of gene expression profiles (RNA-seq) of 19 paracancerous tissue samples and 414 BLCA samples and relevant clinicopathological information. The clinical features of 470 samples were downloaded, including age, gender, grade, and together with TNM stage (8th edition, 2016). Sixty-four samples with incomplete clinical information were excluded from the study (see Table 1).

Microarray gene expression profile GSE40355 was acquired from the GEO database (https://www.ncbi.nlm.nih.gov/) as a validation cohort, which included 8 low-grade and 8-high grade BLCA samples. According to the literature [28], 20 m6A RNA regulators were selected for subsequent analysis (see Supplemental Figure 1).

### 2.2. Expression of m6A Hub Genes in BLCA

**Figure 1:**
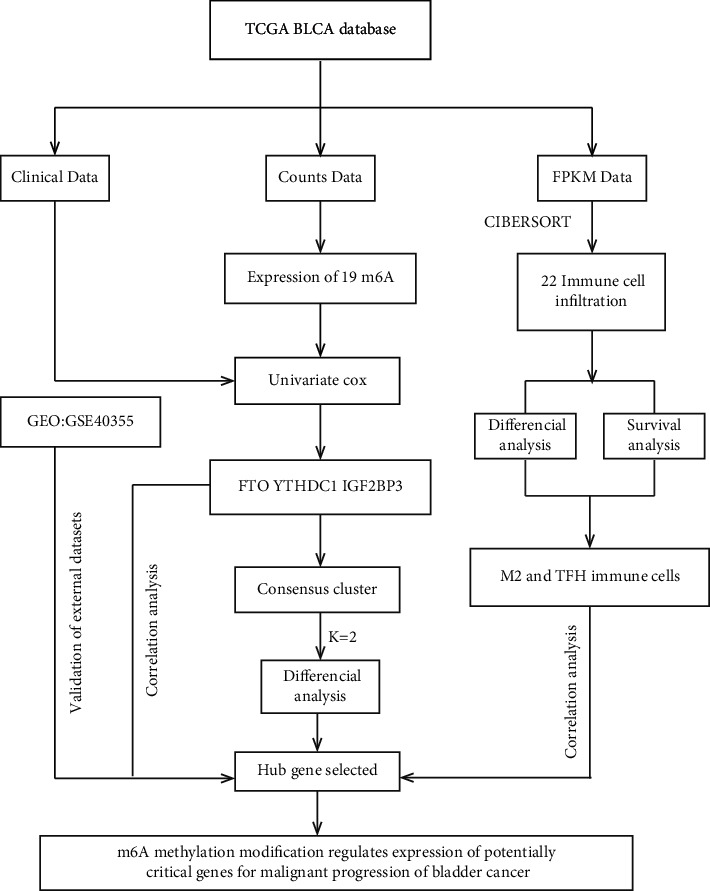
Analysis workflow of this study.

For exploring the underlying effect of m6A genes in BLCA, our study was conducted according to the workflow shown in Figure 1. First, the m6A-related gene expression data in BLCA was used to set up a matrix for subsequent analysis. Then, a violin plot of the expression levels of m6A-related genes in BLCA and paracancerous tissue was drawn using the “vioplot” package in R (RStudio version 3.6.2). Utilizing a univariate Cox regression analysis with a cutoff value of *P* < 0.05, genes with a hazard ratio (HR) >1 or <1 were considered as risk or protective factors. This information was combined with overall survival (OS) for assessing the prognostic value of m6A-related genes. Immunohistochemistry data were funded from the Human Protein Atlas (http://www.proteinatlas.org) to test and verify the protein level of candidate genes in bladder tumor and adjacent normal tissues.

### 2.3. Consensus Clustering Analysis

To further explore the functions of m6A-related genes in BLCA, the “ConsensusClusterPlus” package was used to investigate tumor samples. Consensus clustering, an unsupervised clustering method, is a common classification method for cancer subtype research [29]. The samples can be divided into several subtypes according to different omic datasets, to analyze and compare the subtypes of different diseases. In the study, one hundred iterations were conducted in the clustering process. The consensus clustering number *K* was confirmed by a comprehensive evaluation of the cumulative distribution function of the consensus score, the heat map of the consensus matrix, and the optimal wise consensus pairing value in the clustering.

### 2.4. Differential Analysis and Functional Enrichment Analysis between Subgroups (Cluster 1 and Cluster 2)

For analysis of consensus clustering subgroups, “Limma” package was utilized to identify differentially expressed genes (DEGs). |log2FC| ≥ 1 and FDR <0.05 for filtering the DEGs. Then, functional gene annotation analyses were conducted with the “clusterProfiler” package to functionally annotate the significantly upregulated genes in cluster 1 compared with cluster 2, and the adjusted *P* value <0.05 was considered significant. The Internet-based tools such as Gene Ontology (GO), Kyoto Encyclopedia of Genes and Genomes (KEGG), and gene set enrichment analysis (GSEA) were utilized to uncover molecular mechanisms.

### 2.5. Selection of Hub Genes Associated with Cluster 1

Protein-protein interaction (PPI) network was conducted with STRING (https://string-db.org/) database based on upregulated genes in cluster 1, followed by reconstruction with Cytoscape, version 3.7.1. The “cytoHubba” plug-in and the Matthews correlation coefficient algorithm were used to identify hub genes. Gene expression profiling interactive analysis (GEPIA) (http://gepia.cancer-pku.cn/) was used to determine the impact of these genes on overall survival rate.

### 2.6. Analysis of Immune Infiltration between Subgroups (Cluster 1 and Cluster 2)

We further explored the immune infiltration between BLCA subgroups (cluster 1 and cluster 2). CIBERSORT (https://cibersort.stanford.edu/), a tool from the laboratory of Dr. Ash Alizadeh and developed by Newman et al, was used to analyze the infiltration of 22 kinds of immune cells in BLCA.

### 2.7. Validation of Infiltrated Immune Cells and Hub Genes

To further explore the dynamics of the TME, based on the results of immune infiltration and selected hub genes, we used the Spearman correlation test to calculate the correlation between hub genes and infiltrated immune cells and their corresponding surface markers.

### 2.8. Cell Culture

We used three cell lines (293T, T24, and 5637) for qPCR experimental verification, and all three cell lines were provided by the Laboratory Department of the Third Affiliated Hospital of Kunming Medical University (Yunnan Cancer Hospital). The cells were cultured in RPMI 1640 with 10% FBS,100 U.mL-1 penicillin, and 100 μg.mL-1 streptomycin in a 5% CO_2_ incubator at 37°C. When the cells grow to about 90%, they are digested with 0.05% pancreatin (Biological Industries) in a ratio of 1 : 3 to 1 : 4 for passage.

### 2.9. RNA Extraction and cDNA Synthesis

A cell is collected, RNA with TRIzol reagent is extracted, RNA concentration and purity (A260 nm/280 nm 1.8∼2.1, A260 nm 230 nm > 1.8) are detected under ultra-micro-spectrophotometer, and reverse transcription kit (Roche) is used to synthesize cDNA. After synthesis, cell is stored at −20°C for later use.

### 2.10. Fluorescence Quantitative PCR Amplification

The amplification was carried out according to the instructions of the FastStart Universal SYBR Green Master (Rox) fluorescence quantitative PCR kit. The RT-qPCR system was as follows: FastStart Universal SYBR Green Master (ROX) 10 *μ*L, the upstream and downstream primers each 0.2 *μ*L, the cDNA template 2 *μ*L, dd H2O 7.6 *μ*L, and a total of 20 *μ*L system. The amplification program is as follows: pre-denaturation at 95°C for 30 s, denaturation at 95°C for 5 s, and annealing at 60°C for 30 s, totaling 40 cycles. The 2−ΔCt method (ΔCt = Ct sample-Ct minimum) is used to calculate the relative expression of the target gene. The primer sequence of the target genes is shown in Table 2.

## 3. Results and Discussion

### 3.1. Identifying m6A-Related Genes

**Figure 2:**
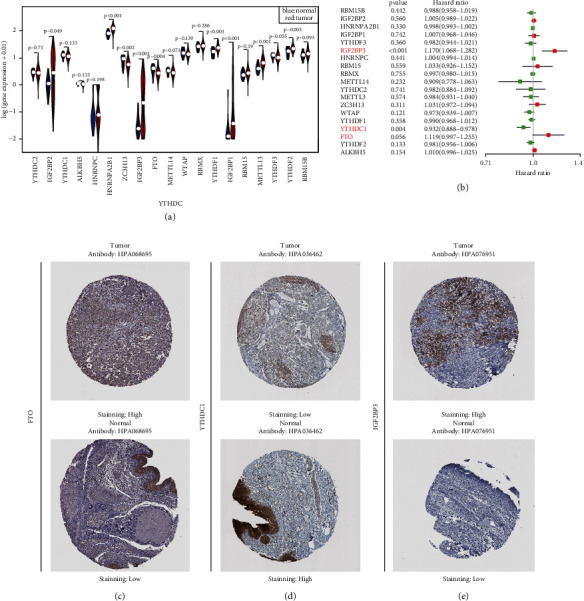
Screening and validation m6A hub methylation regulators in tumor vs. paracarcinoma tissues (a) Violin plot of the m6A RNA methylation regulator expression in tumor vs. adjacent tissue. (b) The hazard ratios (HRs) and 95% confidence intervals (CIs) were calculated by the univariate Cox regression for the 19 m6A regulatory genes in BLCA. (c, e) FTO and IGF2BP3 protein were strongly stained high in bladder tumor tissues compared with paracarcinoma tissues in the Human Protein Atlas database. (d) YTHDC1 protein was stained low in tumor compared with paracarcinoma tissues in the Human Protein Atlas database.

We downloaded information regarding 19 paracancerous tissues and 414 bladder cancer tissue samples from the TCGA database and found that 10 m6A-related genes (ZC3H13, METL3, HNRNPC, HNRNPA2BP1, YTHDF1, YTHDF2, IGF2BP1, IGF2BP2, IGF2BP3, and FTO) were significantly differentially expressed between paracancerous and tumor tissues (see Figure 2(a)). Among them, genes encoding eight regulatory factors (METTL3 (*P* < 0.001), HNRNPC (*P*=0.006), HNRNPA2BP1 (*P* < 0.001), YTHDF1 (*P* < 0.001), YTHDF2 (*P*=0.003), IGF2BP1 (*P* < 0.001), IGF2BP2 (*P*=0.049), and IGF2BP3 (*P* < 0.001)) were upregulated in BLCA, while genes encoding two regulatory factors (ZC3H13 (*P*=0.002) and FTO (*P*=0.004)) were downregulated in BLCA.

Subsequently, the univariate COX regression analysis was conducted to assess the significant factors among these 10 differentially expressed m6A regulatory factors. We found that three m6A regulatory factors were significantly related to OS, including IGF2BP3 (*P* < 0.01), YTHDCI (*P* < 0.05), and FTO (*P*=0.056). Among them, the high expression of FTO (HR = 1.155, 95% CI = 1.035–1.290) and IGF2BP3 (HR = 10175, 95% CI = 1.075–1.285) in BLCA indicates a poor survival rate, while the expression of YTHDC1 (HR = 0.928, 95% CI = 0.885–0.973) was related to better survival (see Figure 2(b)). The Kaplan–Meier plots (see Supplemental Figures 2(a) and 2(c)) further confirmed that the high expression of FTO and IGF2BP3 indicates a poor prognosis for BLCA patients, while the high expression of YTHDC1 correlates with better OS. Notably, immunohistochemistry staining data revealed that the levels of FTO and IGF2BP3 proteins were significantly higher in BLCA (see Figure 2(c)), while the level of YTHDC1 protein was lower in BLCA relative to paracancerous tissue (see Figure 2(c)).

### 3.2. Correlation Analysis between the FTO, IGF2BP3, and YTHDC1 and Clinical Characteristics

As is shown in Supplemental Figures 3(a) and 3(b), FTO and IGF2BP3 were found highly expressed in patients with BLCA that was high grade, stage III or IV, and T stage III or IV, while the expression of YTHDC1 was significantly lower in patients that were younger than 65 years and patients who died from BLCA (see Supplemental Figure 3(c)), which suggests that m6A-related genes may be involved in the malignant progression of BLCA tumors.

### 3.3. Identification of Two Clusters of BLCA according to Consensus Clustering Analysis of FTO, IGF2BP3, and YTHDC1

**Figure 3:**
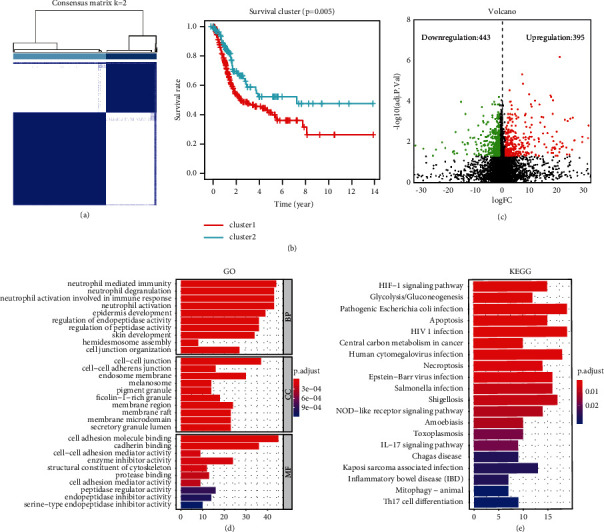
Consensus clustering analysis and subgroup difference analysis. (a) Consensus clustering matrix for BLCA. (b) Overall survival rates of two subgroups. *P*=0.005 (cluster 1 vs. cluster 2). (c) Volcano map of DEGs between cluster 1 and cluster 2. (d) GO enrichment analysis of 395 upregulation genes in cluster 1. (e) KEGG enrichment analysis of 395 upregulation genes in cluster 1.

Based on the 3 selected m6A genes, we performed consensus clustering on gene expression in the TCGA BLCA datasets. The K value represents the number of cluster analysis subgroups. According to the results of the empirical cumulative distribution function and the selection criteria of the clustering value K (see Supplemental Figures 4(a) and 4(b)), we found that when *K* = 2, the interference between groups was the smallest, so the BLCA cohort was divided into two different subgroups, cluster 1 and cluster 2 (see Figure 3(a)). To further verify the appropriateness of our classification, “PCA” was used to perform principal component analysis with RStudio version 3.5.1 to verify the reliability of the subgroups. The results (see Supplemental Figure 4(c)) showed that the two subgroups could be distinguished very well, and the survival curve (see Figure 3(b)) also showed that the OS of cluster 1 was considerably lower than that of cluster 2 (*P*=0.005).

### 3.4. Differential Expression Analysis and Functional Enrichment of Subgroups

Next, we conducted differential expression analysis between the two subgroups (cluster 1 vs. cluster 2). The volcano diagram (see Figure 3(c)) identified a total of 838 DEGs (fold change >1 or < −1, *P* < 0.05), of which 395 were significantly upregulated (fold change >1, *P* < 0.05) and 443 were downregulated (fold change <−1, *P* < 0.05). Further, GO and KEGG were performed on the 395 upregulated genes. The GO functional annotation results (Figure 3(d)) showed that upregulated genes were mainly enriched in tumor malignant progression pathways, such as neutrophil-mediated immunity, neutrophil activation involved in immune response, cell-cell adhesion mediator activity, and cell adhesion mediator activity. KEGG results (see Figure 3(d)) showed that upregulated genes were abundantly expressed in immune-related signaling pathways, such as apoptosis, IL-17 signaling pathways, and Th17 cell differentiation.

### 3.5. Hub Genes Are Highly Related to Regulators of the m6A Modification

**Figure 4:**
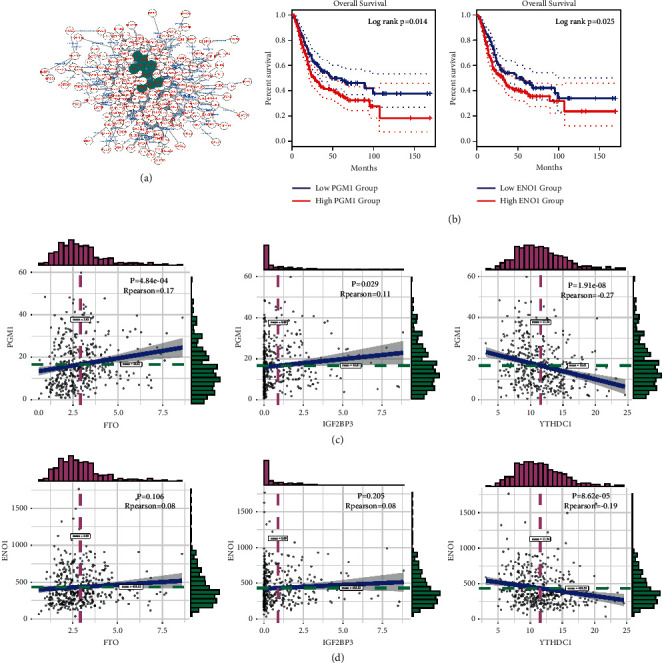
Screening hub genes and analysis of its correlation with m6A methylation regulators. (a) STRING for protein interaction analysis in cluster 1. Green represents the screening of 10 hub genes. (b) Overall survival rates of two hub genes (PGM1 and ENO1) in BLCA. (c) The relationship of PGM1 with indicated m6A genes (FTO, IGF2BP3, and YTHDC1). (d) The relationship of ENO1 with indicated m6A genes (FTO, IGF2BP3, YTHDC1).

STRING is a well-known database to predict protein-protein interactions (PPIs). In this study, we inputted 395 significantly upregulated genes into the STRING online analysis tool to analyze the interaction relationships between their proteins and then imported the obtained data into Cytoscape to visualize the PPI network. We utilized the cytoHubba app to obtain ten hub genes: ENO1, GAPDH, LDHA, PGAM1, PGK1, PKM, SLC2A1, SOD2, and TPI1 (see Figure 4(a)). Then, we used GEPIA2 to perform survival analysis on the 10 hub genes and found that only PGM1 and ENO1 were associated with a significant survival rate (see Supplemental Figures 5(a)–5(h)) (see Figure 4(b)).

Next, we investigated the expression of PGM1, ENO1, and m6A-related genes to identify the potential mechanism of aberrant upregulation in BLCA. The correlation analysis exhibited that the expression of PGM1, ENO1, and m6A-related genes was highly correlated (see Figures 4(c) and 4(d)). In particular, the expression levels of PGM1 and ENO1 were significantly positively correlated with those of FTO and IGF2BP3 and significantly negatively correlated with that of YTHDC1, suggesting that m6A-related genes regulate the expression of key genes PGM1 and ENO1, thus regulating the occurrence and development of BLCA.

### 3.6. Distinct Immune Infiltration Analysis of the Two Clusters

**Figure 5:**
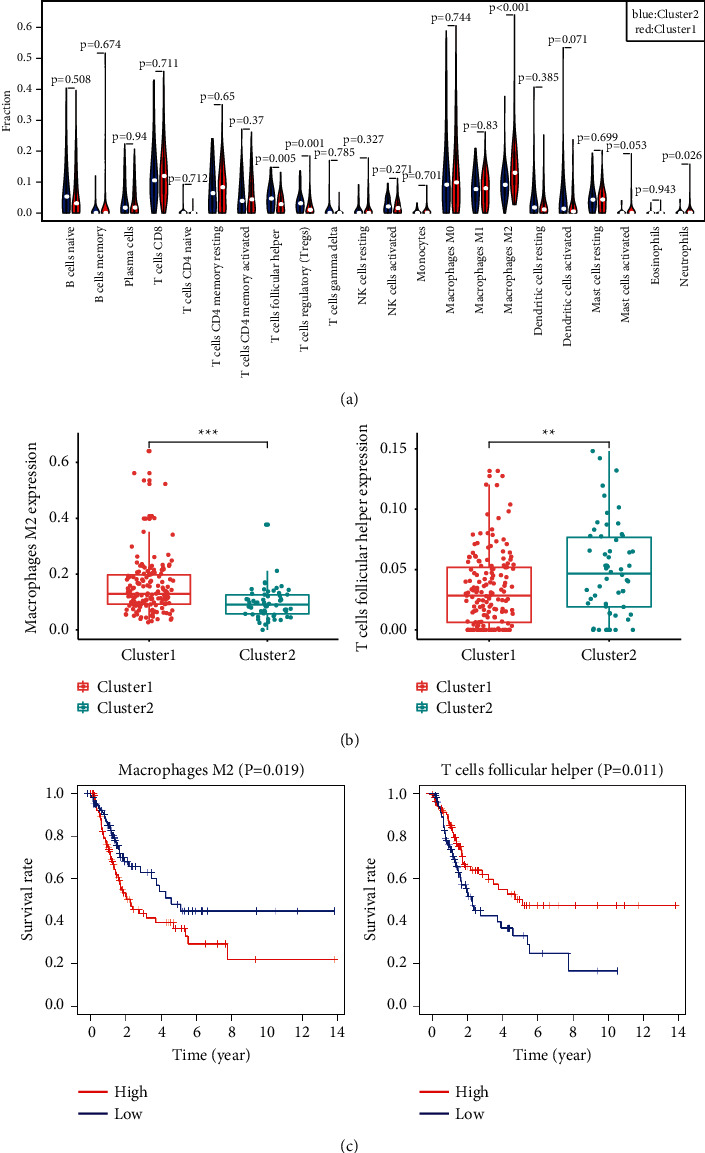
Distinct immune infiltration analysis of two clusters. (a) Violin plot of 22 infiltrated immune cells in different clusters, and the Wilcoxon rank-sum was used for the significance test. (b) Histogram shows the expression of M2 macrophages and TFH cells in different subgroups. ^*∗*^*P* < 0.05, ^*∗∗*^*P* < 0.01, and ^*∗∗∗*^*P* < 0.001. (c) Overall survival rates of M2 macrophages and TFH cells in BLCA.

Through the above gene function annotation, it was found that the upregulated genes in cluster 1 were highly enriched in immunologically related pathways. Therefore, we speculated that the malignant progression of BLCA was related to its immune microenvironment. To explore immune infiltration in bladder tumors, we retrieved a matrix of infiltration of 22 kinds of immune cells in 215 BLCA patients (*P* < 0.05) by a deconvolution method, CIBERSORT. We used the “vioplot” package to visualize the infiltration of 22 immune cells in cluster 1 and cluster 2 (see Figure 5(a)) and found that the infiltration of M2 (*P* < 0.001), neutrophils (*P*=0.026), follicular helper T (TFH) cells (*P*=0.005), and Treg (*P*=0.001) was significantly different between the two subgroups. The histogram (see Figure 5(b)) showed that M2 macrophage cells were highly expressed in cluster 1, while TFH cells were highly expressed in cluster 2. Survival analysis (see Figure 5(c)) showed that high infiltration of M2 predicts a poor survival rate, while high infiltration of TFH cells predicts a better survival rate.

### 3.7. Hub Genes (PGM1 and ENO1) Are Significantly Related to Immune Infiltration

**Figure 6:**
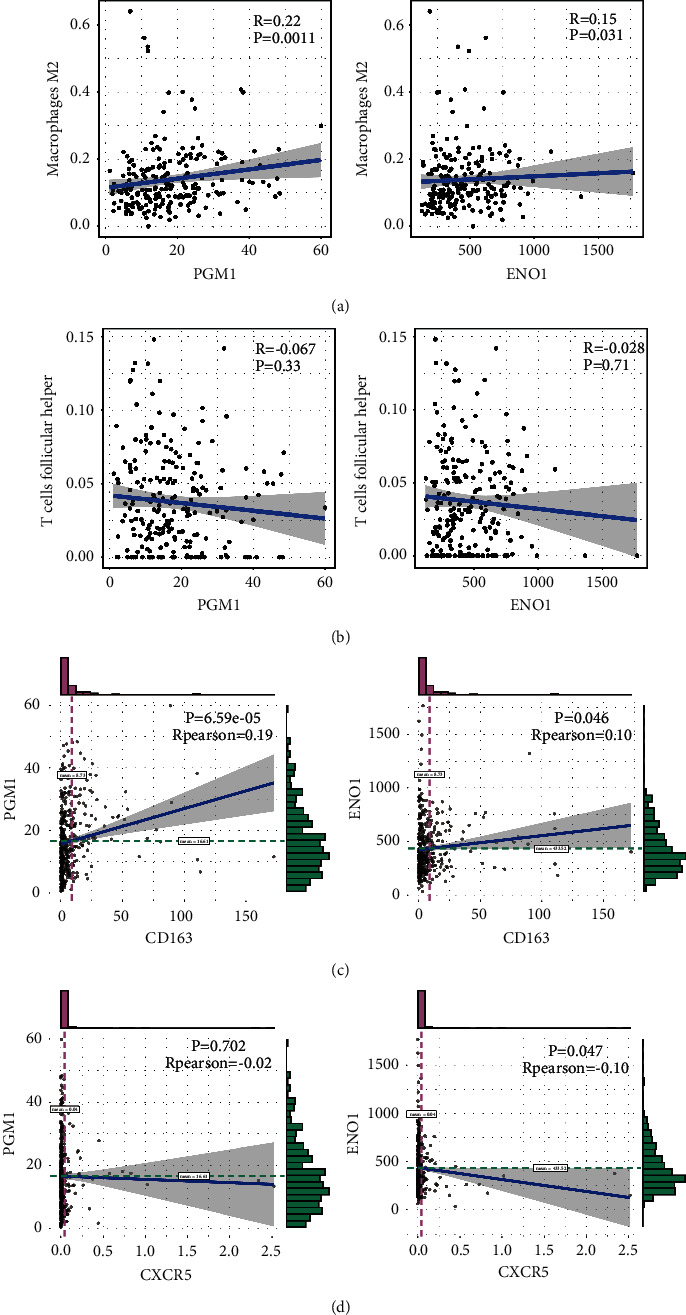
High correlation of hub genes with immune cells and their markers. (a) The relationship of PGM1 and ENO1 with macrophage 2. (b) The relationship of PGM1 and ENO1 with TFH. (c) The relationship of PGM1 and ENO1 with macrophage 2 marker CD163. (d) The relationship of PGM1 and ENO1 with TFH marker CXCR5.

The Spearman method was used to explore the potential connections between PGM1 and ENO1 and immune infiltrating cells in BLCA. Interestingly, we found that the hub genes PGM1 and ENO1 were positively correlated with M2 macrophage and significantly negatively correlated with TFH cells (see Figures 6(a) and 6(b)). Accordingly, PGM1 and ENO1 were also significantly positively correlated with the M2 surface marker CD163 but significantly negatively correlated with the TFH cell marker CXCR5 (see Figures 6(c) and 6(d)). These data indicate that the hub genes PGM1 and ENO1 are significantly related to immune infiltration.

Meanwhile, we used the same method to explore the relationship between gene encoding regulators of the m6A modification (FTO, IGF2BP3, YTHDC1) and immune infiltration in BLCA. Interestingly, we also found a significant positive correlation between FTO and IGF2BP3 and M2 macrophage infiltration, while YTHDC1 was negatively correlated with M2 macrophage infiltration and positively correlated with TFH cell infiltration (see Supplemental Figures 6(a)–6(f)).

### 3.8. GSEA of Single Gene and Validation of Hub Genes

**Figure 7:**
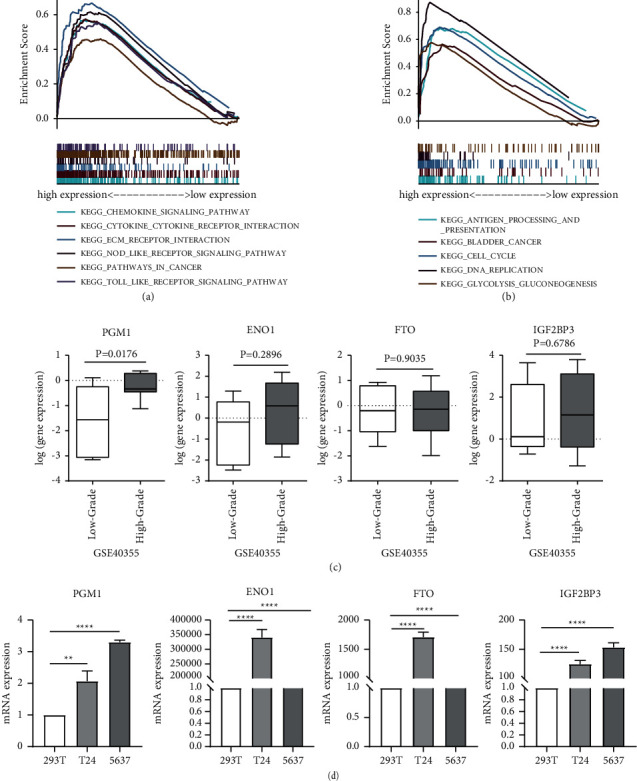
GSEA of single gene and validation of hub genes. (a, b) The most involved significant pathways that PGM1 and ENO1-related genes enriched in. (c) The hub gene expression in GSE40355 datasets. (d) The hub gene expression in 293T, T24, and 5637 cell lines.

The hallmark analysis in GSEA was conducted for PGM1 and ENO1. The results showed that the pathways involving PGM1 include the chemokine signaling pathway, cytokine-cytokine receptor interaction pathway, extracellular matrix receptor intersection pathway, and nucleotide oligomerization domain-like receptor signaling pathway (see Figure 7(a)). The most significant pathways involving ENO1 include the bladder cancer pathway, cell cycle pathway, DNA replication, and glycolysis and gluconeogenesis pathway (see Figure 7(b)). To verify the reliability of the results, we downloaded eight low-grade and eight high-grade BLCA samples from the NCBI GEO database GSE40355 (Table 3). The differential analysis was utilized to explore the expression differences in key genes PGM1, ENO1, IGF2BP3, and FTO between the high-grade and low-grade groups. As shown in Figure 7(c), PGM1 was expressed at a significantly higher level in the high-grade sample (*P*=0.0176), as was ENO1 (*P*=0.2896), IGF2BP3 (*P*=0.6786), and FTO (*P*=0.9036). Fluorescence quantitative PCR results also showed that PGM1, ENO1, FTO, and IGF2BP3 all showed significantly high expression in human bladder cancer cells (T24, 5637) (see Figure 7(d)).

## 4. Discussion

The global incidence of BLCA is increasing every year, and its mortality rate is gradually rising [30, 31]. Therefore, it is important to explore the pathogenesis of BLCA and to find better therapeutic targets. With the rapid development of omics, a variety of high-throughput tumor databases, including TCGA and GEO, have been established, providing supporting data for analysis of the occurrence and progression of tumors [32].

Our study was dealt mainly with the changes in regulators of the m6A modification in the TCGA database, combined with information regarding immune infiltration to explore potential effective markers in the malignant progression of BLCA. M6A is the most common modification of mRNA, and increasing numbers of researches have shown that it is connected with cell proliferation, differentiation, invasion, and metastasis of tumors [33, 34]. Weng et al. [35] found that abnormalities of the m6A modification are closely associated with the prevalence and development of hematological malignancies. In addition, the inhibition of ZNF217-dependent m6A methylation of NANOG and KLF4 mRNA was found to be enhanced in breast cancer cells under hypoxia, thus promoting the occurrence and development of breast cancer. Therefore, the abnormal expression of m6A RNA methylation regulators in tumor tissues may provide a new target for the development of antitumor drugs and may provide a potential biomarker for the molecular diagnosis of tumors.

Through identification of the characteristics of the expression of the three hub regulators of the m6A modification (FTO, IGF2BP3, and YTHDC1) and using the consensus clustering analysis, BLCA samples in the TCGA database were divided into two subgroups. Combined with survival and prognosis information, cluster 1 became the object of our study. We comprehensively analyzed the genes that were differentially expressed between the two subgroups, and two hub genes (PGM1 and ENO1) related to prognosis were ultimately selected. We found that the high expression of ENO1 in bladder tumor samples predicted a worse survival rate, and the correlation analysis showed that there was a significant positive correlation between the expression of FTO and IGF2BP3, suggesting that ENO1 plays a key role in the malignant progression group (cluster 1) of BLCA.

Alpha-enolase, which is encoded by the ENO1 gene, is a subtype of enolase, a key enzyme in glycolysis [36]. It catalyzes the conversion of 2-phosphoglycerate into phosphoenolpyruvate. On the surface of cancer cells, ENO1 acts as a plasminogen receptor, promoting the degradation of plasminogen to plasmin, a serine protease involved in the degradation of extracellular matrix, thus facilitating the invasion and metastasis of the cell [37]. Relevant researches have shown that ENO1 is highly expressed in varieties of tumors types and is involved in tumor angiogenesis, invasion, and metastasis. For example, in pancreatic cancer, silencing ENO1 inhibited the migration and invasion of pancreatic ductal adenocarcinoma cells in vitro and in vivo [37, 38]. Cheng et al. [39] uncovered that the expression of ENO1 in colorectal cancer tissues is significantly correlated with clinicopathological factors such as lymph node infiltration and TNM stage and is positively correlated with the high expression of RAB1A; the co-overexpression of both ENO1 and RAB1A was associated with poor prognosis in colorectal cancers. In non-small cell lung cancer, Xu et al. conducted TGF-*β*-1-induced EMT experiments and EGF-stimulated ERK1/2 activation and other related experiments confirmed that ENO1 inhibits ERK1/2 phosphorylation to inhibit the EMT process, thereby inhibiting tumor development and metastasis [40]. In breast cancer, researchers found that C5aR1-positive neutrophils secrete IL-1-*β* and TNF-*α* and they cooperatively activate ERK1/2 signal and phosphorylate WTAP at serine 341 to stabilize WTAP protein. As m6A methyltransferase, WTAP promotes RNA m6A methylation of ENO1 and affects the glycolysis activity of breast cancer cells, thereby affecting tumor progression and metastasis [41]. In our study, we first divided the bladder cancer samples into two subgroups based on three key m6A methylation gene expression values using the consensus clustering analysis method. After differential analysis, we found that ENO1 was highly expressed in the malignant samples of bladder cancer, suggesting that ENO1 may be regulated by m6A methylation in bladder cancer and further speculated that the ENO1 gene can induce the activity of its enzyme to affect the glycolysis activity of bladder cancer cells, thereby affecting the progression of the cancer of the bladder.

Similarly, we also found that PGM1 was significantly highly expressed in malignant BLCA, and its high expression indicated a worse survival rate, demonstrating that it may be a potential biomarker for malignant progression of BLCA. The enzyme coded by PGM1 gene, phosphoglucomutase, is essential to the process of carbohydrate biosynthesis and metabolism. It plays an important role in glycogen synthesis by catalyzing the bidirectional mutual conversion of D-glucose 1-phosphate and D-glucose 6-phosphate [42]. Clinical studies have shown that mutations within the PGM1 are associated with an inborn error in metabolism previously classified as a glycogen storage disease, and a defect in PGM1 has recently been shown to be a congenital glycation disease [43]. Recently, the role of PGM1 in cancer has also been widely reported. Through immunohistochemical analysis of a large number of hepatocellular carcinoma (HCC) patients, Jin et al. [44] found that PGM1 was underexpressed in HCC compared with paracancerous tissue and was correlated with the degree of malignancy and poor prognosis of HCC. The low expression of PGM1 inhibited the glycogen synthesis pathway of tumor cells, making glucose more available for glycolysis, thus promoting tumor cell proliferation and the malignant progression of HCC. In another study [45], glucose deprivation activated AMP-activated protein kinase-dependent HDAC8 phosphorylation in lung cancer, triggering the elevated expression of PGM1 and promoting the malignant progression of lung cancer, suggesting that PGM1 is a promising anticancer therapeutic target. In our study, PGM1 was significantly highly expressed in patients with high-grade bladder cancer, and its high expression seriously affected the prognosis of patients. Further, we also found the m6A methylation-related genes FTO, IGF2BP3, and YTHDC1 might participate in the process of PGM1 methylation modification, thereby affecting tumor progression and metastasis.

CIBERSORT was used to analyze the immune infiltration of the two subgroups. It was found that M2 was significantly more infiltrated in cluster 1 with rapid malignant progression, and survival curves showed that high infiltration predicted a worse survival rate. As is becoming more well known, the genesis and development of tumors are affected by the TME, and among various inflammatory cells infiltrating into the TME, tumor-associated macrophages occupy the main component [8, 46]. Studies have shown that M2-type tumor-associated macrophages tend to be highly infiltrated in various tumor tissues and that this infiltration is associated with poor prognosis [47]. After stimulation by various cytokines, such as IL-10 and TGF-*α*, M2 macrophages activate a Th2-type immune response and promote the secretion of anti-inflammatory cytokines, such as IL-10 and TGF-*α* and chemokines including C-C motif chemokine ligand (CCL) 17, CCL18, CCL22, and CCL24, thus promoting the occurrence and development of tumors [48, 49]. In addition, M2 macrophages also inhibit the function of CD8^+^ T cells and impede the efficacy of cancer chemoradiotherapy, leading to tumor progression and poor prognosis [50].

Here, the correlation analysis demonstrated that the elevated expression of ENO1 and PGM1 was positively correlated with infiltration of M2 macrophages and the presence of its surface marker CD163, which further suggests that ENO1 and PGM1 may be potential biomarkers for the malignant progression of BLCA. Finally, from the GEO validation set GSE40335, which included eight low-grade and eight high-grade BLCA samples, ENO1 and PGM1 were relatively highly expressed in high-grade BLCA samples, further verifying the significant role of ENO1 and PGM1 in the malignant progression of BLCA.

## 5. Conclusions

This research uncovered relationships between the expression of regulators of m6A methylation and immune infiltration in BLCA and identified PGM1 and ENO1 as genes that are highly correlated with the malignant progression of BLCA. These genes may be effective indicators for the prediction of the malignant progression of BLCA. Our findings can provide clues to the production and effects of m6A-RNA methylation in BLCA and lay a solid foundation for the next steps of m6A-RNA methylation research.

## Figures and Tables

**Table 1:** Clinical feature information of patients.

TCGA cohort		
Characteristic	Number	%
Age		
<65	151	37.1
>=65	256	62.9

Sex		
Female	106	26
Male	301	74

Status		
Alive	250	61.4
Dead	157	38.6

Stage		
I-II	131	32.2
III-IV	274	67.3
Unknown	2	0.5
Grade		
High grade	384	94.3
Low grade	20	4.9
Unknown	3	0.8

T stage		
T1-2	41	10.1
T3-4	53	13
T2a-T2b	81	19.9
T3a-Tb	151	37.1
T4a-Tb	49	12
Unknown	32	7.9

N stage		
N0-1	282	69.3
N2-3	83	20.4
NX	36	8.8
Unknown	6	1.5

M stage		
M0	195	48.3
M1	11	2.6
MX	198	48.6
Unknown	3	0.8

TCGA: The Cancer Genome Atlas.

**Table 2:** Primer sequence of target genes.

Gene	Sequence (5′-3′)	Length
PGM1	F:CCTCCTTCATGTAAAACCTG	20
	R:GTTAAGACCAAGGCGTATCA	20

ENO1	F:CAGGCCAATGGTTGGGGCGT	20
	R:GGCTTGCCTGCCCACAGCTT	20

FTO	F: TGGGTTCATCCTACAACGG	19
	R: CCTCTTCAGGGCCTTCAC	18

IGF2BP3	F:AGTTGTTGTCCCTCGACC	18
	R:AGCCTTCTGTTGTTGGTGCT	20

**Table 3:** Detailed information of patients in GSE40355 cohorts.

	GSE40355 cohorts			
Accession	Tissue	Gender	Age	Stage
Low grade				
GSM991931	Low-grade papillary urothelial carcinoma	Male	59	pTaG1
GSM991932	Low-grade papillary urothelial carcinoma	Male	65	pTaG2
GSM991933	Low-grade papillary urothelial carcinoma	Male	70	pTaG2
GSM991934	Low-grade papillary urothelial carcinoma	Male	76	pTaG2
GSM991935	Low-grade papillary urothelial carcinoma	Male	63	pTaG1
GSM991936	Low-grade papillary urothelial carcinoma	Male	75	pTaG2
GSM991937	Low-grade papillary urothelial carcinoma	Male	77	pTaG2
GSM991938	Low-grade papillary urothelial carcinoma	Male	79	pTaG2

High grade				
GSM991939	High-grade papillary urothelial carcinoma	Male	71	pT2G3
GSM991940	High-grade papillary urothelial carcinoma	Female	76	pT2rG3
GSM991941	High-grade papillary urothelial carcinoma	Male	75	pT1G3
GSM991942	High-grade papillary urothelial carcinoma	Female	73	pT2G3
GSM991943	High-grade papillary urothelial carcinoma	Male	74	pT2aG3
GSM991944	High-grade papillary urothelial carcinoma	Male	62	pT1G3
GSM991945	High-grade papillary urothelial carcinoma	Male	74	pT1G3
GSM991946	High-grade papillary urothelial carcinoma	Male	73	pT1G3

## Data Availability

All data were obtained from TCGA data portal (https://portal.gdc.cancer.gov/) using the Genomic Data Commons Data Transfer Tool. The cohort consists of gene expression profiles (RNA-seq) of 19 paracancerous tissue samples and 414 BLCA samples and relevant clinicopathological information. Microarray gene expression profile GSE40355 was acquired from the GEO database (https://www.ncbi.nlm.nih.gov/) as a validation cohort, which included 8 low-grade and 8 high-grade BLCA samples.

## Supplementary Materials

Supplementary Materials Supplemental Figure 1: classification of 20 methylation regulators. Supplemental Figure 2: validation of 3 hub m6A regulators. (a) Overall survival rate of FTO in BLCA. (b) Overall survival rate of IGF2BP3 in BLCA. (c) Overall survival rate of YTHDC1 in BLCA. Supplemental Figure 3: correlation of 3 m6A hub methylation regulators with clinicopathological characteristics. (a) Distribution of FTO expression in grade, stage, T, and fustat. *P*=0.0036, 0.00078, 0.0071, and 0.011, respectively, by Kruskal–Wallis rank-sum *t*. (b) Distribution of IGF2BP3 expression in grade, stage, T, and fustat. *P*=2.7*e* − 07, 0.0036, 0.0012, and 0.0017, respectively, by Kruskal–Wallis rank-sum *t*. (c) Distribution of YTHDC1 expression in age and fustat. *P*=0.01 and 0.078, respectively, by Kruskal–Wallis rank-sum *t*. Supplemental Figure 4: consensus clustering analysis based on 3 m6A hub methylation regulators vs. 19 m6A methylation regulators. (a) Consensus clustering distribution function (CDF) for bladder cancer. (b) Relative changes in the area under the CDF curve for bladder cancer. (c) Principal component analysis (PCA) for total RNA expression pattern. Subgroups are marked with blue and red. (d) Consensus clustering matrix for BLCA based on 19 m6A methylation regulators. (e) The Kaplan–Meier OS curves for the two subgroups. *P*=0.155 (cluster 1 vs. cluster 2). (f) Principal component analysis (PCA) for total RNA expression pattern. Subgroups are marked with blue and red. Supplemental Figure 5: survival analysis of 10 hub genes in cluster 1. (a–h) Overall survival rates of 8 hub genes in cluster 1. (a) TPL1, *P*=0.062. (b) SOD2, *P*=0.57. (c) SLC2A1, *P*=0.35. (d) PKM, *P*=0.15. (e) PGK1, *P*=0.46. (f) PGAM1, *P*=0.093. (g) LDHA, *P*=0.067. (h) GAPDH, *P*=0.092. Supplemental Figure 6: 3 m6A hub methylation regulators are significantly related to immune infiltration. (a–c) The relationship of FTO, IGF2BP3, and YTHDC1 with macrophage 2. (d–f) The relationship of FTO, IGF2BP3, and YTHDC1 with TFH.
