# Supplementary figures and images for: PGM1 and ENO1 Promote the Malignant Progression of Bladder Cancer via Comprehensive Analysis of the m6A Signature and Tumor Immune Infiltration
Source: J Oncol. 2022 Feb 24;2022:8581805. doi: 10.1155/2022/8581805 (PMC8894041; doi:10.1155/2022/8581805)

# m6A RNA methylation regulators

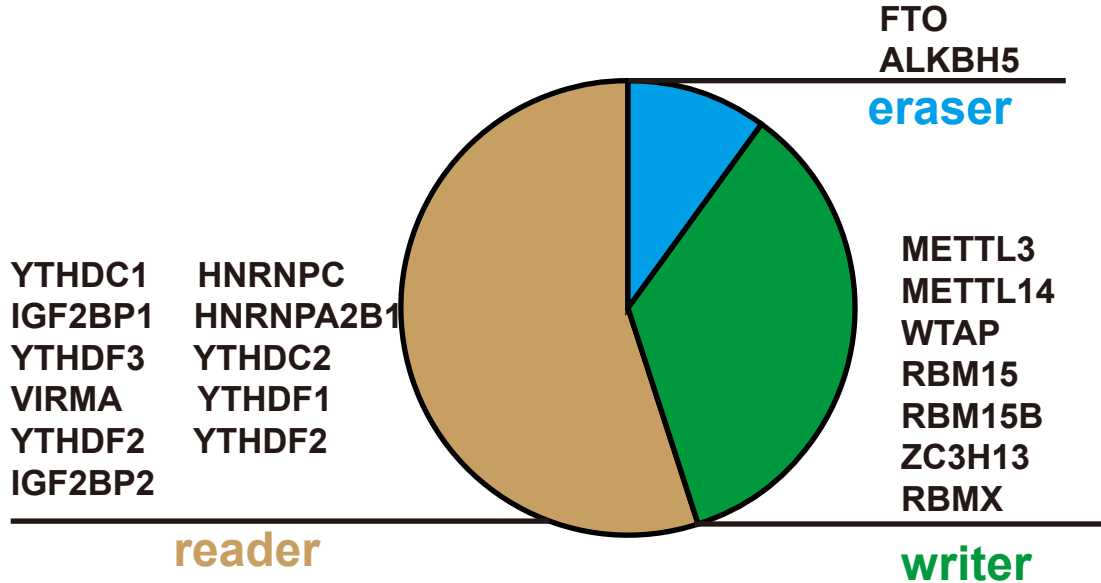

Supplement: Supplementary Materials — Supplemental Figure 1: classification of 20 methylation regulators. Supplemental Figure 2: validation of 3 hub m6A regulators. (a) Overall survival rate of FTO in BLCA. (b) Overall survival rate of IGF2BP3 in BLCA. (c) Overall survival rate of YTHDC1 in BLCA. Supplemental Figure 3: correlation of 3 m6A hub methylation regulators with clinicopathological characteristics. (a) Distribution of FTO expression in grade, stage, T, and fustat. P=0.0036, 0.00078, 0.0071, and 0.011, respectively, by Kruskal–Wallis rank-sum t. (b) Distribution of IGF2BP3 expression in grade, stage, T, and fustat. P=2.7e − 07, 0.0036, 0.0012, and 0.0017, respectively, by Kruskal–Wallis rank-sum t. (c) Distribution of YTHDC1 expression in age and fustat. P=0.01 and 0.078, respectively, by Kruskal–Wallis rank-sum t. Supplemental Figure 4: consensus clustering analysis based on 3 m6A hub methylation regulators vs. 19 m6A methylation regulators. (a) Consensus clustering distribution function (CDF) for bladder cancer. (b) Relative changes in the area under the CDF curve for bladder cancer. (c) Principal component analysis (PCA) for total RNA expression pattern. Subgroups are marked with blue and red. (d) Consensus clustering matrix for BLCA based on 19 m6A methylation regulators. (e) The Kaplan–Meier OS curves for the two subgroups. P=0.155 (cluster 1 vs. cluster 2). (f) Principal component analysis (PCA) for total RNA expression pattern. Subgroups are marked with blue and red. Supplemental Figure 5: survival analysis of 10 hub genes in cluster 1. (a–h) Overall survival rates of 8 hub genes in cluster 1. (a) TPL1, P=0.062. (b) SOD2, P=0.57. (c) SLC2A1, P=0.35. (d) PKM, P=0.15. (e) PGK1, P=0.46. (f) PGAM1, P=0.093. (g) LDHA, P=0.067. (h) GAPDH, P=0.092. Supplemental Figure 6: 3 m6A hub methylation regulators are significantly related to immune infiltration. (a–c) The relationship of FTO, IGF2BP3, and YTHDC1 with macrophage 2. (d–f) The relationship of FTO, IGF2BP3, and YTHDC1 with TFH. [file 8581805.f1.zip › 8581805.f1/Supplemental1.pdf]

**A****FTO**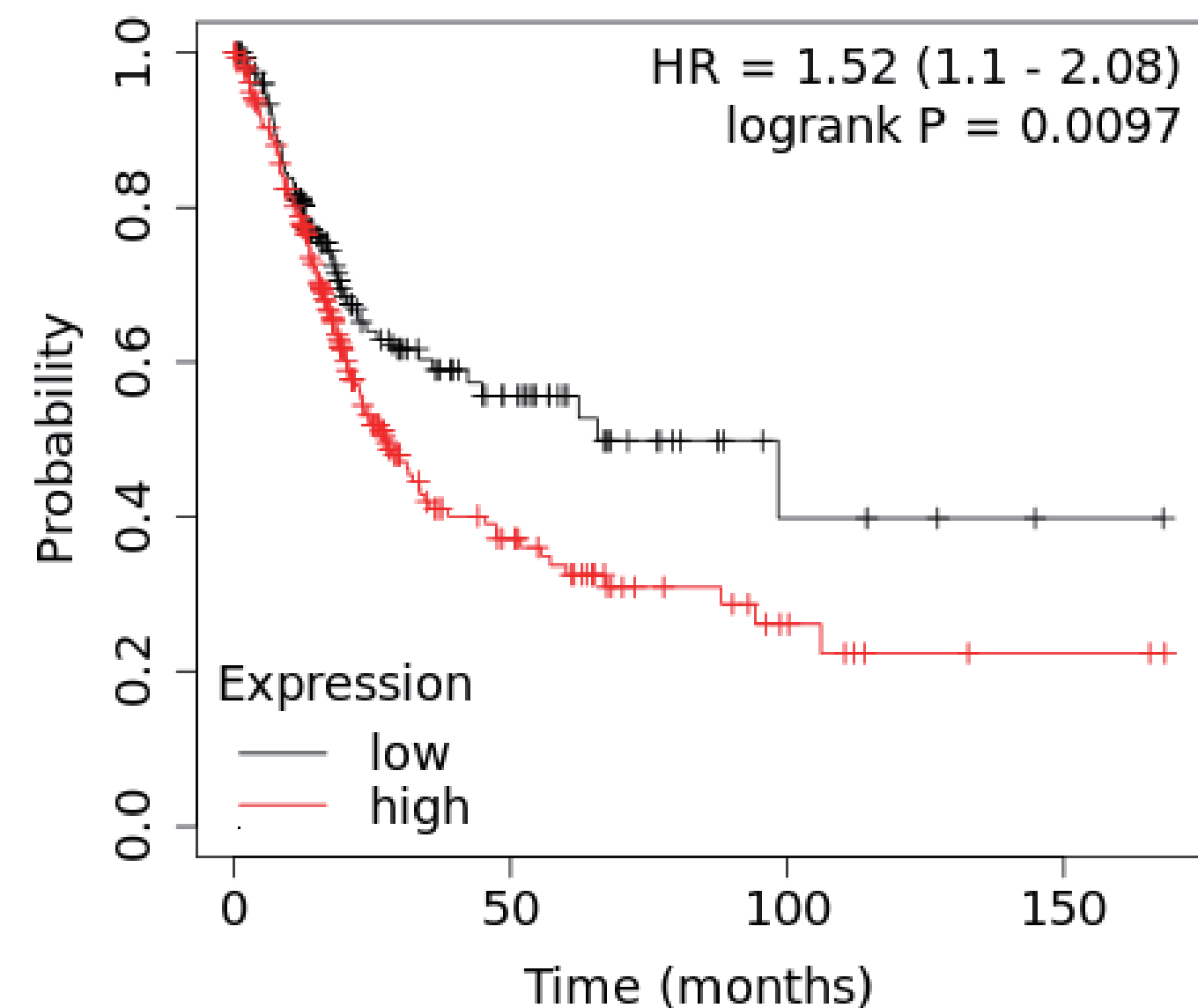

Number at risk

| low | high |
|-----|------|
| 160 | 244  |
| 30  | 36   |
| 4   | 8    |
| 1   | 2    |

**B****YTHDC1**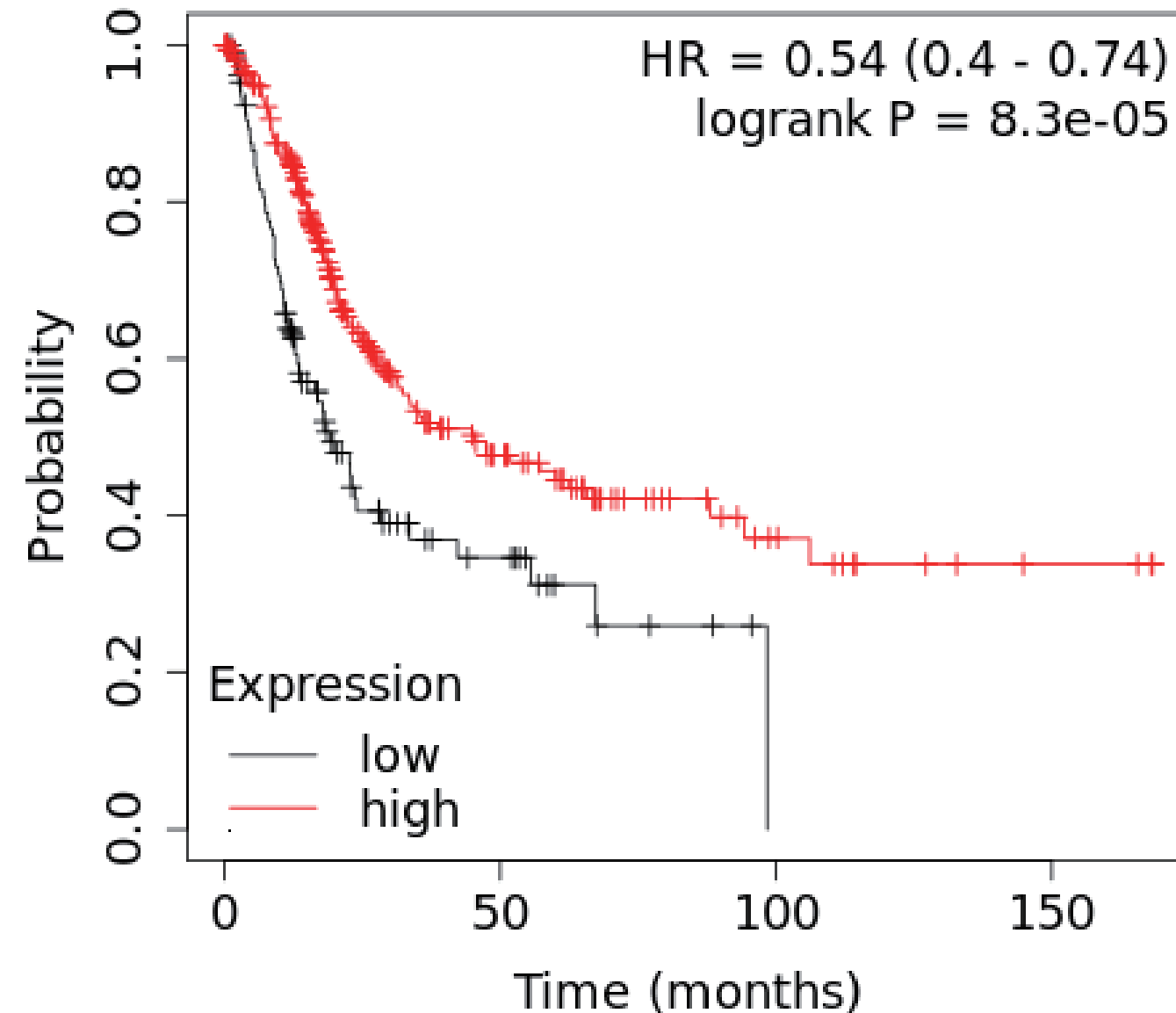

Number at risk

| low | high |
|-----|------|
| 109 | 295  |
| 14  | 52   |
| 0   | 12   |
| 0   | 3    |

**C****IGF2BP3**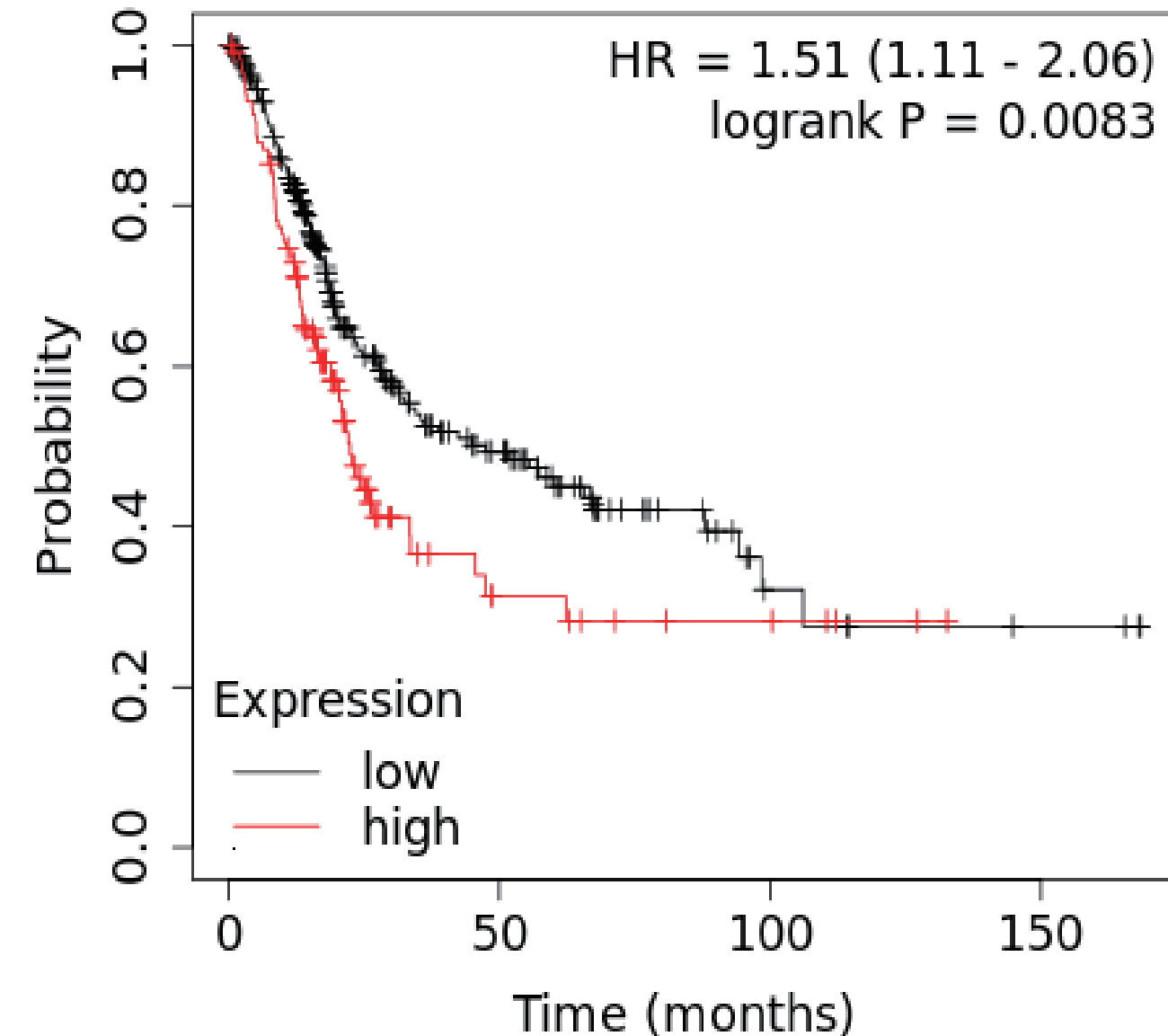

Number at risk

| low | high |
|-----|------|
| 287 | 117  |
| 56  | 10   |
| 7   | 5    |
| 3   | 0    |

Supplement: Supplementary Materials — Supplemental Figure 1: classification of 20 methylation regulators. Supplemental Figure 2: validation of 3 hub m6A regulators. (a) Overall survival rate of FTO in BLCA. (b) Overall survival rate of IGF2BP3 in BLCA. (c) Overall survival rate of YTHDC1 in BLCA. Supplemental Figure 3: correlation of 3 m6A hub methylation regulators with clinicopathological characteristics. (a) Distribution of FTO expression in grade, stage, T, and fustat. P=0.0036, 0.00078, 0.0071, and 0.011, respectively, by Kruskal–Wallis rank-sum t. (b) Distribution of IGF2BP3 expression in grade, stage, T, and fustat. P=2.7e − 07, 0.0036, 0.0012, and 0.0017, respectively, by Kruskal–Wallis rank-sum t. (c) Distribution of YTHDC1 expression in age and fustat. P=0.01 and 0.078, respectively, by Kruskal–Wallis rank-sum t. Supplemental Figure 4: consensus clustering analysis based on 3 m6A hub methylation regulators vs. 19 m6A methylation regulators. (a) Consensus clustering distribution function (CDF) for bladder cancer. (b) Relative changes in the area under the CDF curve for bladder cancer. (c) Principal component analysis (PCA) for total RNA expression pattern. Subgroups are marked with blue and red. (d) Consensus clustering matrix for BLCA based on 19 m6A methylation regulators. (e) The Kaplan–Meier OS curves for the two subgroups. P=0.155 (cluster 1 vs. cluster 2). (f) Principal component analysis (PCA) for total RNA expression pattern. Subgroups are marked with blue and red. Supplemental Figure 5: survival analysis of 10 hub genes in cluster 1. (a–h) Overall survival rates of 8 hub genes in cluster 1. (a) TPL1, P=0.062. (b) SOD2, P=0.57. (c) SLC2A1, P=0.35. (d) PKM, P=0.15. (e) PGK1, P=0.46. (f) PGAM1, P=0.093. (g) LDHA, P=0.067. (h) GAPDH, P=0.092. Supplemental Figure 6: 3 m6A hub methylation regulators are significantly related to immune infiltration. (a–c) The relationship of FTO, IGF2BP3, and YTHDC1 with macrophage 2. (d–f) The relationship of FTO, IGF2BP3, and YTHDC1 with TFH. [file 8581805.f1.zip › 8581805.f1/Supplemental2.pdf]

**A**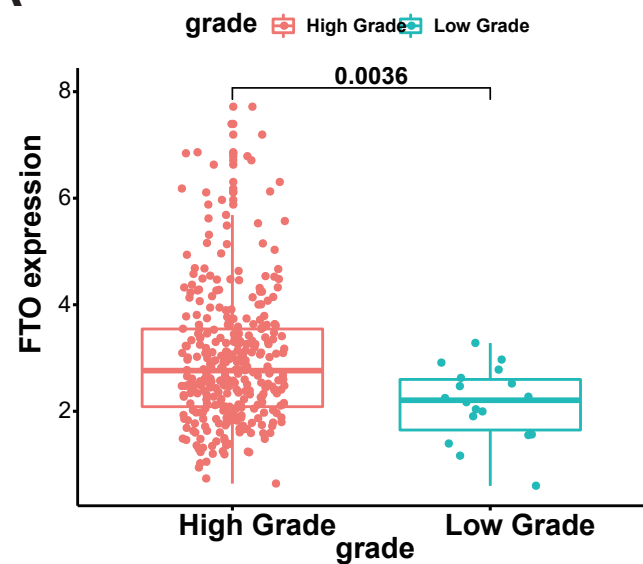**B**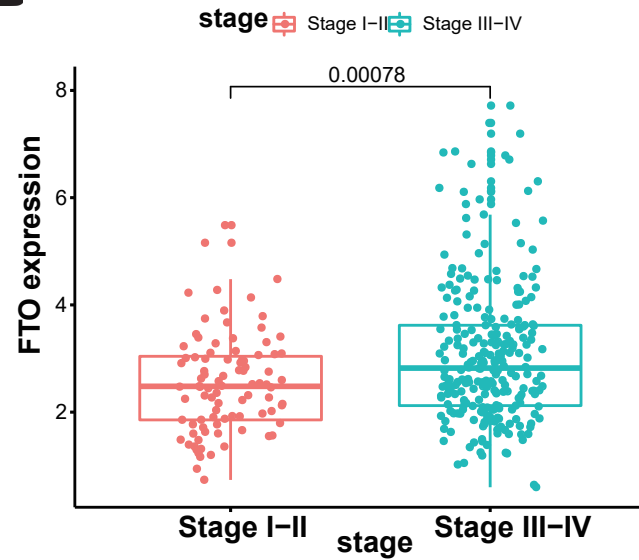**C**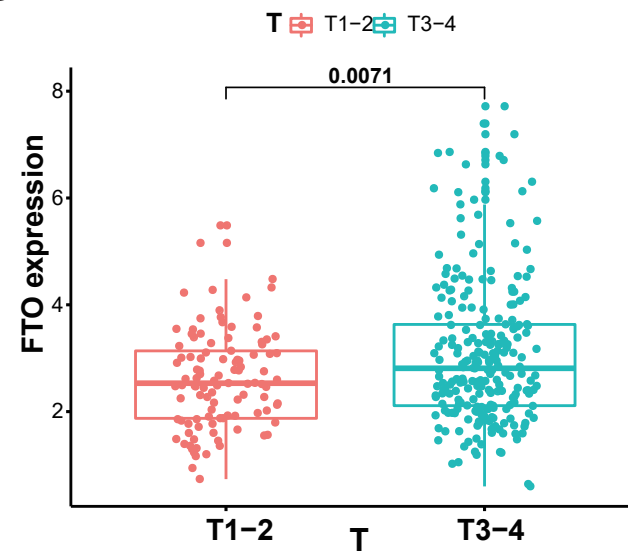**D**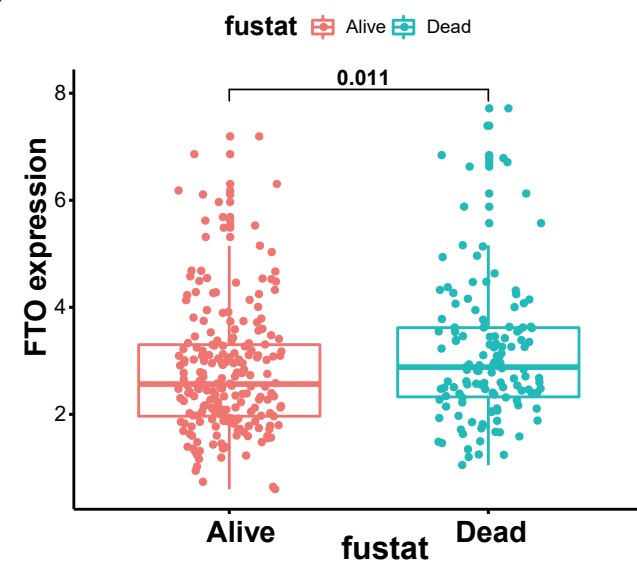**E**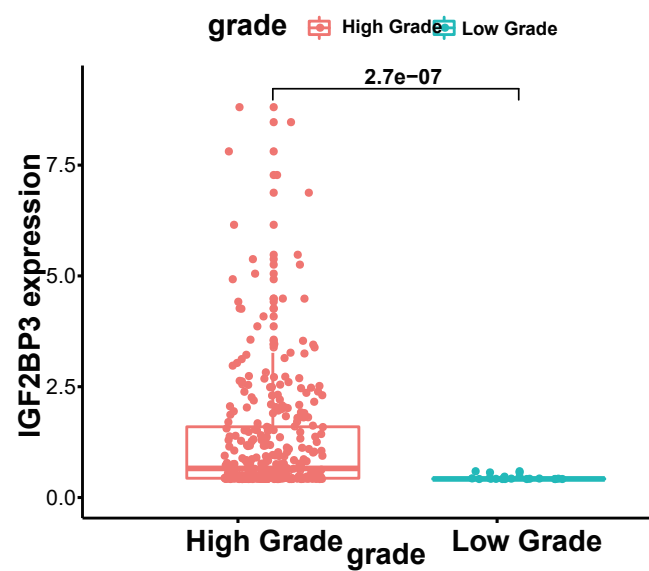**F**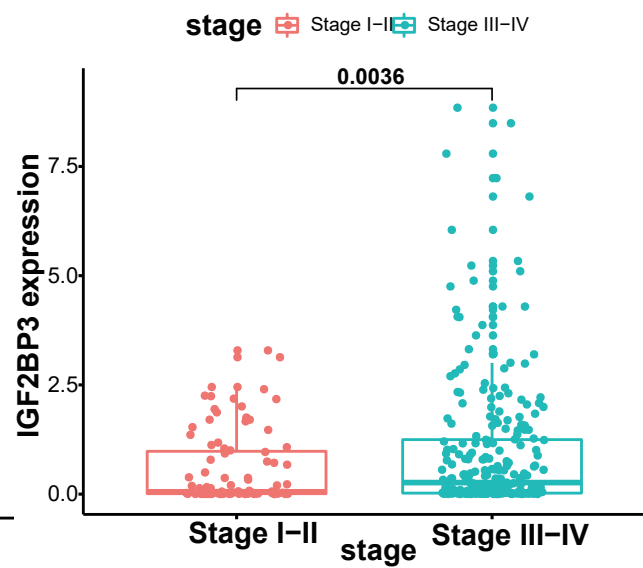**G**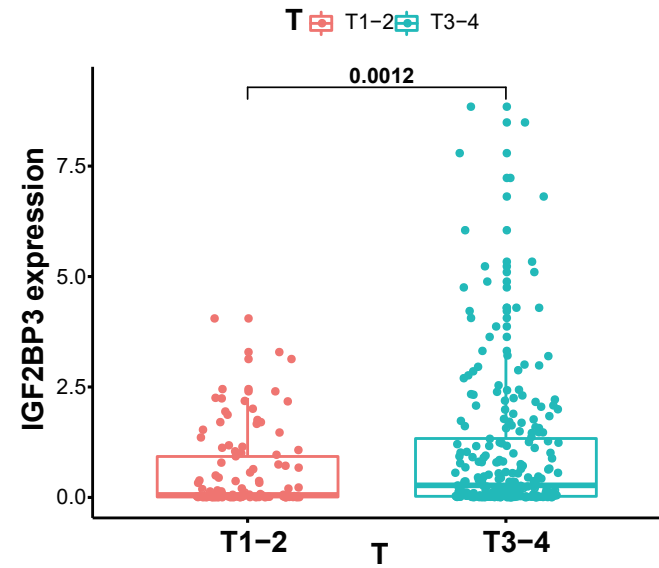**H**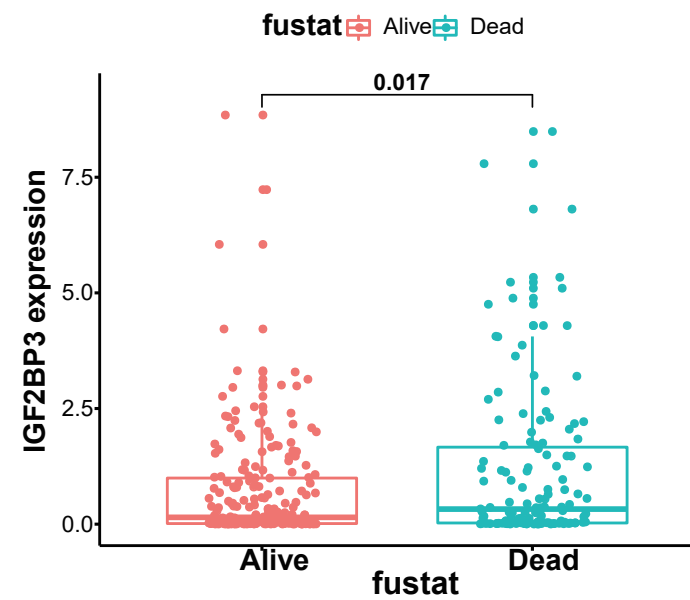**I**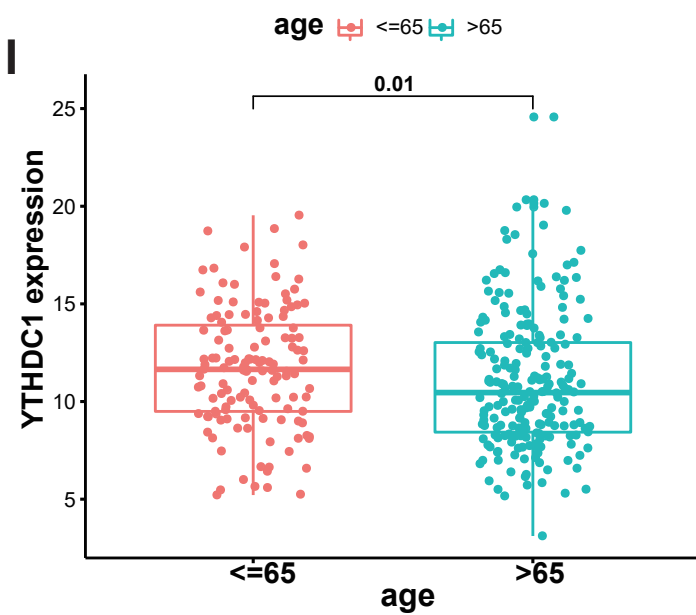**J**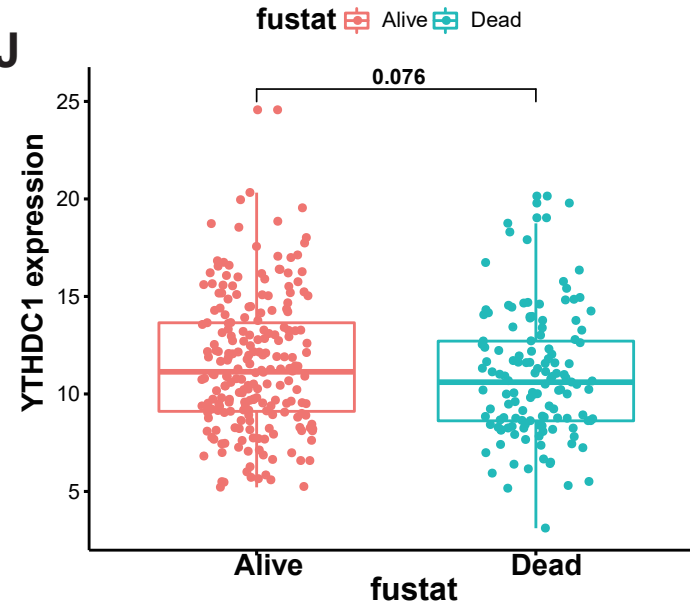

Supplement: Supplementary Materials — Supplemental Figure 1: classification of 20 methylation regulators. Supplemental Figure 2: validation of 3 hub m6A regulators. (a) Overall survival rate of FTO in BLCA. (b) Overall survival rate of IGF2BP3 in BLCA. (c) Overall survival rate of YTHDC1 in BLCA. Supplemental Figure 3: correlation of 3 m6A hub methylation regulators with clinicopathological characteristics. (a) Distribution of FTO expression in grade, stage, T, and fustat. P=0.0036, 0.00078, 0.0071, and 0.011, respectively, by Kruskal–Wallis rank-sum t. (b) Distribution of IGF2BP3 expression in grade, stage, T, and fustat. P=2.7e − 07, 0.0036, 0.0012, and 0.0017, respectively, by Kruskal–Wallis rank-sum t. (c) Distribution of YTHDC1 expression in age and fustat. P=0.01 and 0.078, respectively, by Kruskal–Wallis rank-sum t. Supplemental Figure 4: consensus clustering analysis based on 3 m6A hub methylation regulators vs. 19 m6A methylation regulators. (a) Consensus clustering distribution function (CDF) for bladder cancer. (b) Relative changes in the area under the CDF curve for bladder cancer. (c) Principal component analysis (PCA) for total RNA expression pattern. Subgroups are marked with blue and red. (d) Consensus clustering matrix for BLCA based on 19 m6A methylation regulators. (e) The Kaplan–Meier OS curves for the two subgroups. P=0.155 (cluster 1 vs. cluster 2). (f) Principal component analysis (PCA) for total RNA expression pattern. Subgroups are marked with blue and red. Supplemental Figure 5: survival analysis of 10 hub genes in cluster 1. (a–h) Overall survival rates of 8 hub genes in cluster 1. (a) TPL1, P=0.062. (b) SOD2, P=0.57. (c) SLC2A1, P=0.35. (d) PKM, P=0.15. (e) PGK1, P=0.46. (f) PGAM1, P=0.093. (g) LDHA, P=0.067. (h) GAPDH, P=0.092. Supplemental Figure 6: 3 m6A hub methylation regulators are significantly related to immune infiltration. (a–c) The relationship of FTO, IGF2BP3, and YTHDC1 with macrophage 2. (d–f) The relationship of FTO, IGF2BP3, and YTHDC1 with TFH. [file 8581805.f1.zip › 8581805.f1/Supplemental3.pdf]

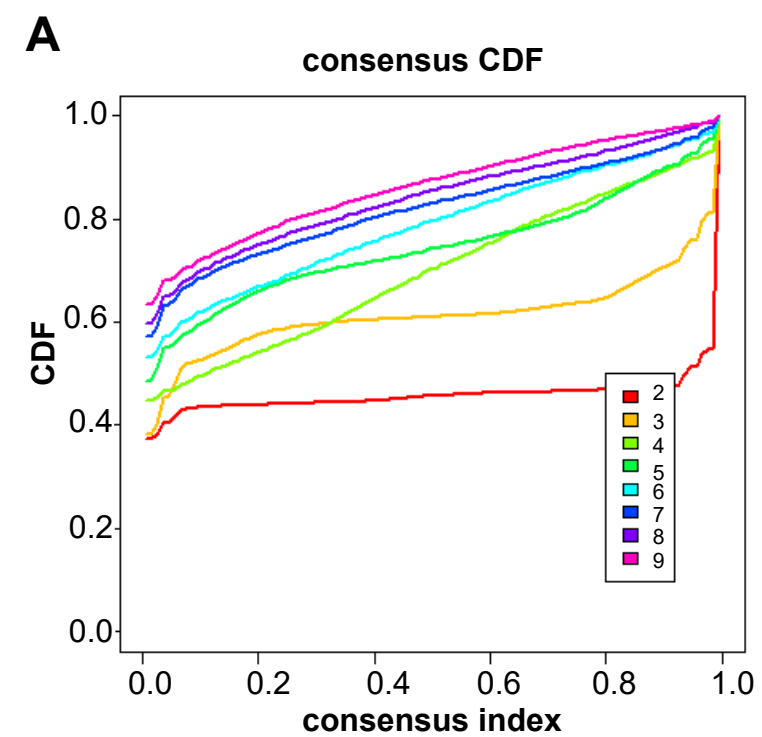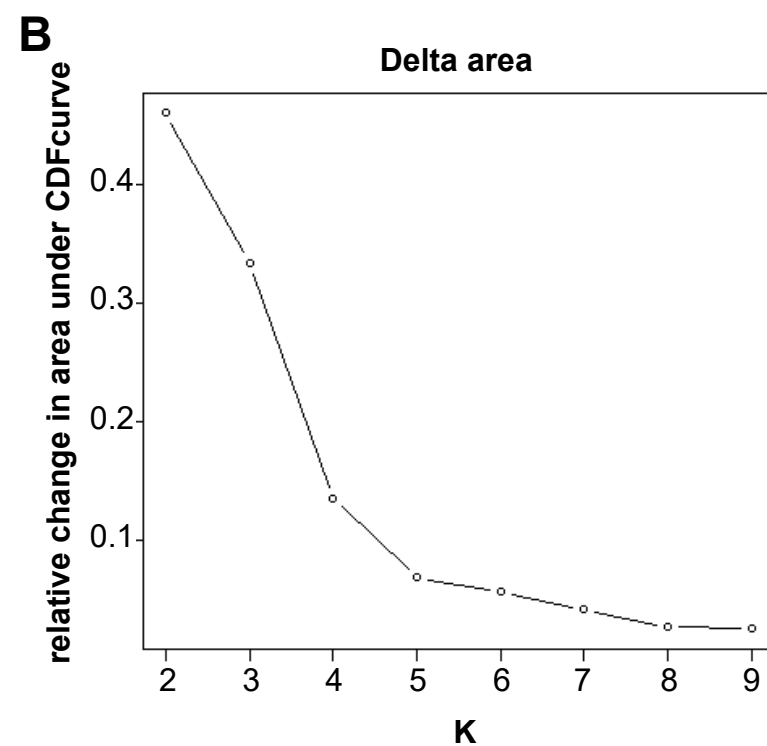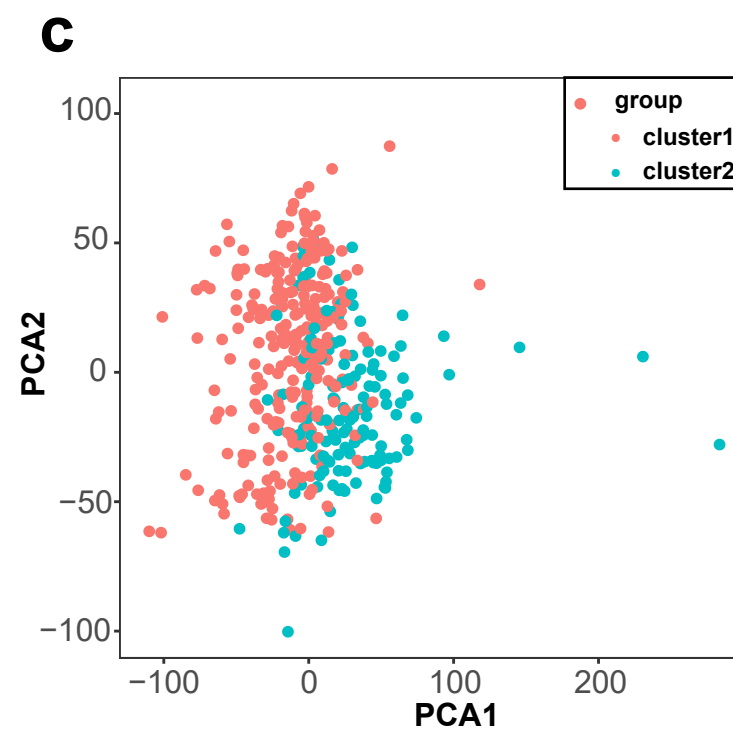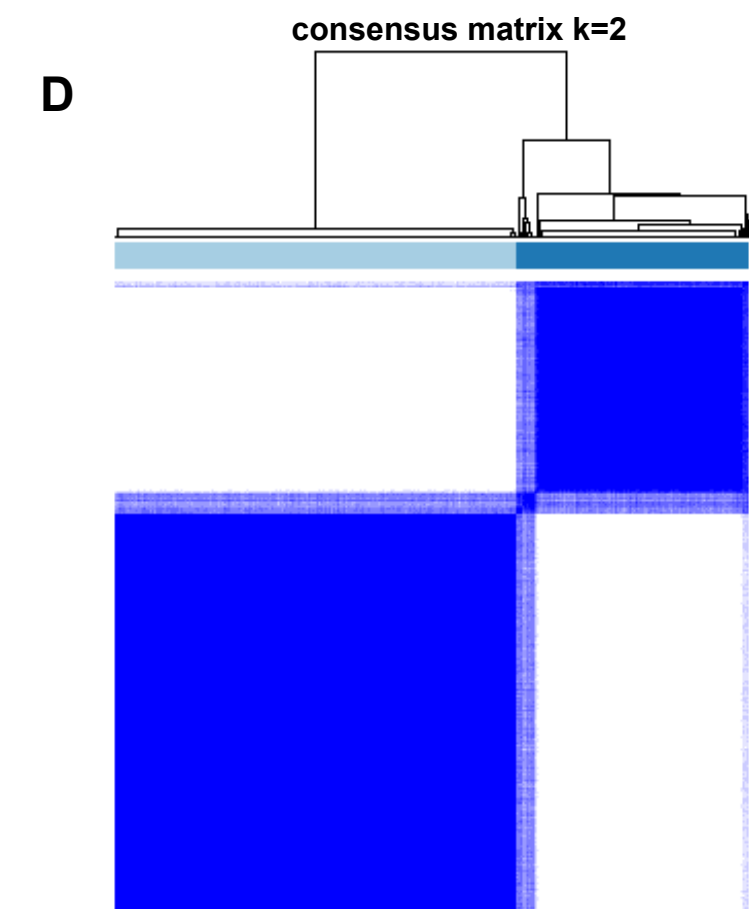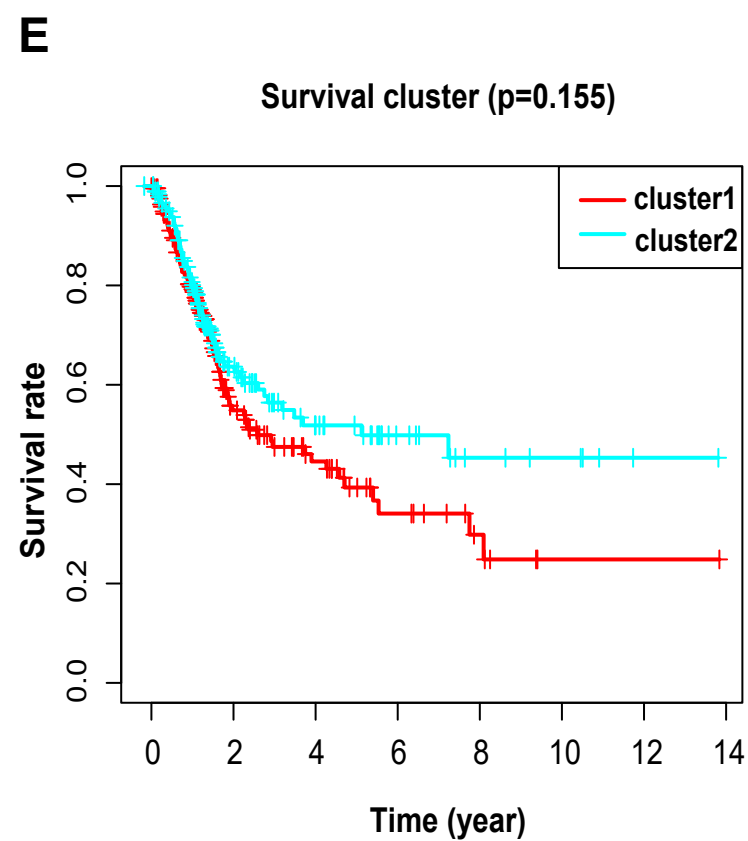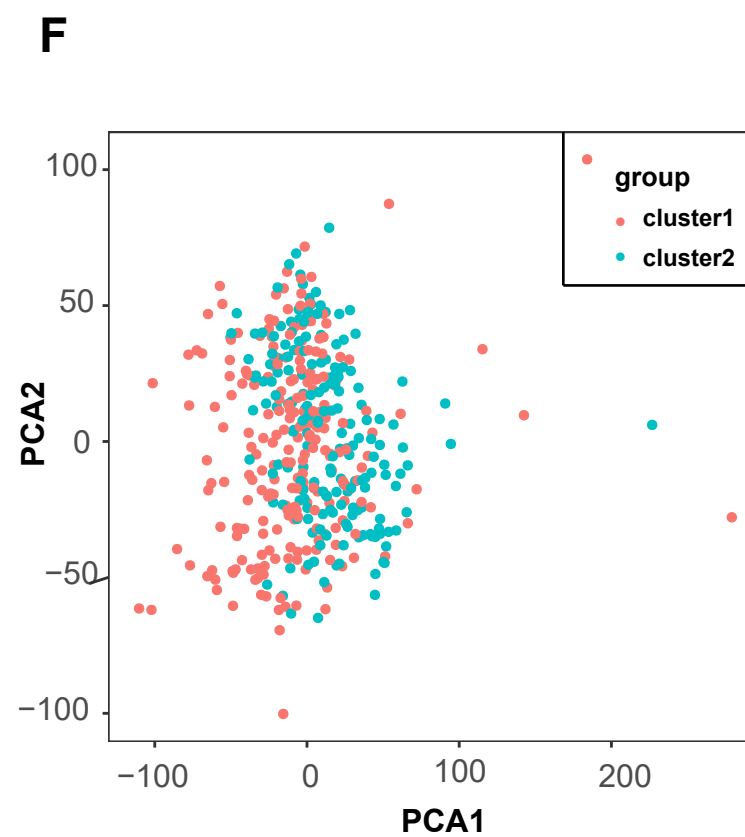

Supplement: Supplementary Materials — Supplemental Figure 1: classification of 20 methylation regulators. Supplemental Figure 2: validation of 3 hub m6A regulators. (a) Overall survival rate of FTO in BLCA. (b) Overall survival rate of IGF2BP3 in BLCA. (c) Overall survival rate of YTHDC1 in BLCA. Supplemental Figure 3: correlation of 3 m6A hub methylation regulators with clinicopathological characteristics. (a) Distribution of FTO expression in grade, stage, T, and fustat. P=0.0036, 0.00078, 0.0071, and 0.011, respectively, by Kruskal–Wallis rank-sum t. (b) Distribution of IGF2BP3 expression in grade, stage, T, and fustat. P=2.7e − 07, 0.0036, 0.0012, and 0.0017, respectively, by Kruskal–Wallis rank-sum t. (c) Distribution of YTHDC1 expression in age and fustat. P=0.01 and 0.078, respectively, by Kruskal–Wallis rank-sum t. Supplemental Figure 4: consensus clustering analysis based on 3 m6A hub methylation regulators vs. 19 m6A methylation regulators. (a) Consensus clustering distribution function (CDF) for bladder cancer. (b) Relative changes in the area under the CDF curve for bladder cancer. (c) Principal component analysis (PCA) for total RNA expression pattern. Subgroups are marked with blue and red. (d) Consensus clustering matrix for BLCA based on 19 m6A methylation regulators. (e) The Kaplan–Meier OS curves for the two subgroups. P=0.155 (cluster 1 vs. cluster 2). (f) Principal component analysis (PCA) for total RNA expression pattern. Subgroups are marked with blue and red. Supplemental Figure 5: survival analysis of 10 hub genes in cluster 1. (a–h) Overall survival rates of 8 hub genes in cluster 1. (a) TPL1, P=0.062. (b) SOD2, P=0.57. (c) SLC2A1, P=0.35. (d) PKM, P=0.15. (e) PGK1, P=0.46. (f) PGAM1, P=0.093. (g) LDHA, P=0.067. (h) GAPDH, P=0.092. Supplemental Figure 6: 3 m6A hub methylation regulators are significantly related to immune infiltration. (a–c) The relationship of FTO, IGF2BP3, and YTHDC1 with macrophage 2. (d–f) The relationship of FTO, IGF2BP3, and YTHDC1 with TFH. [file 8581805.f1.zip › 8581805.f1/Supplemental4.pdf]

**A**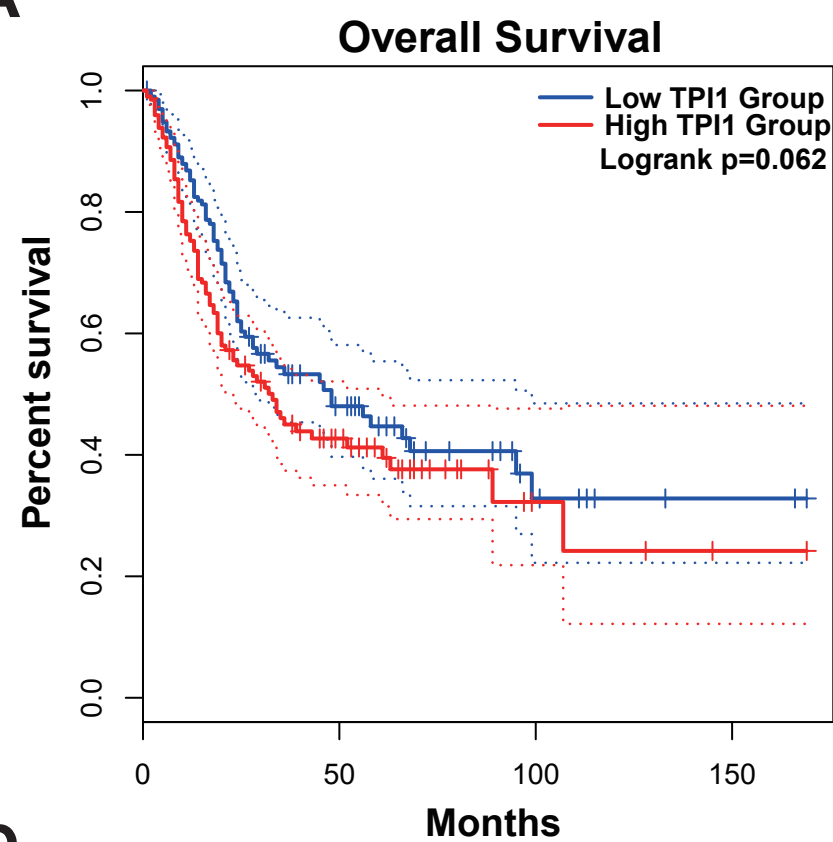**B**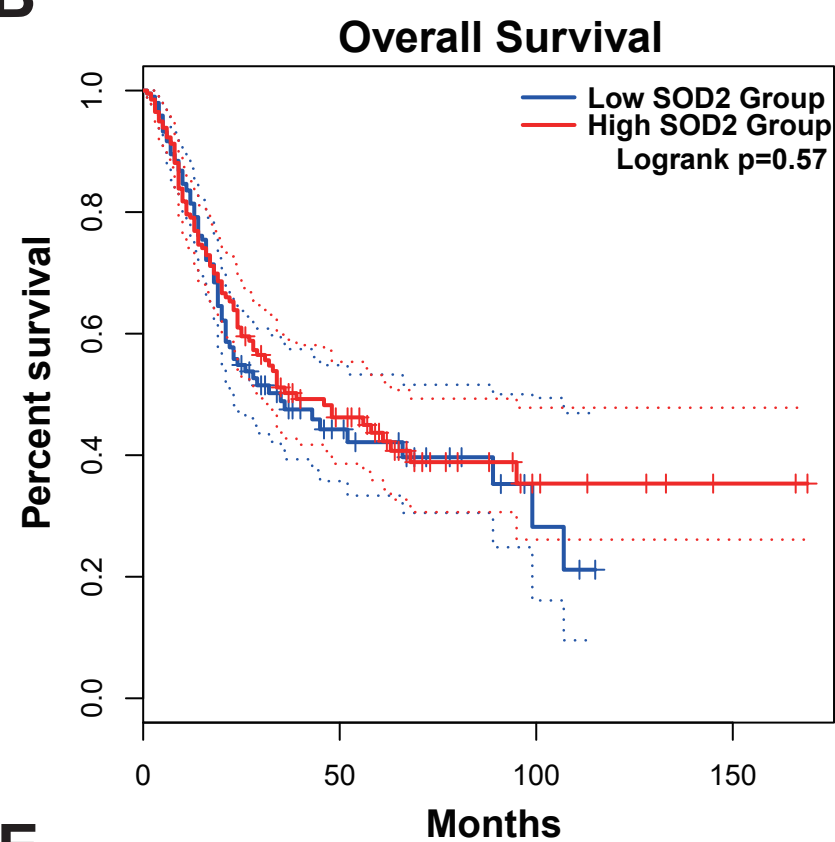**C**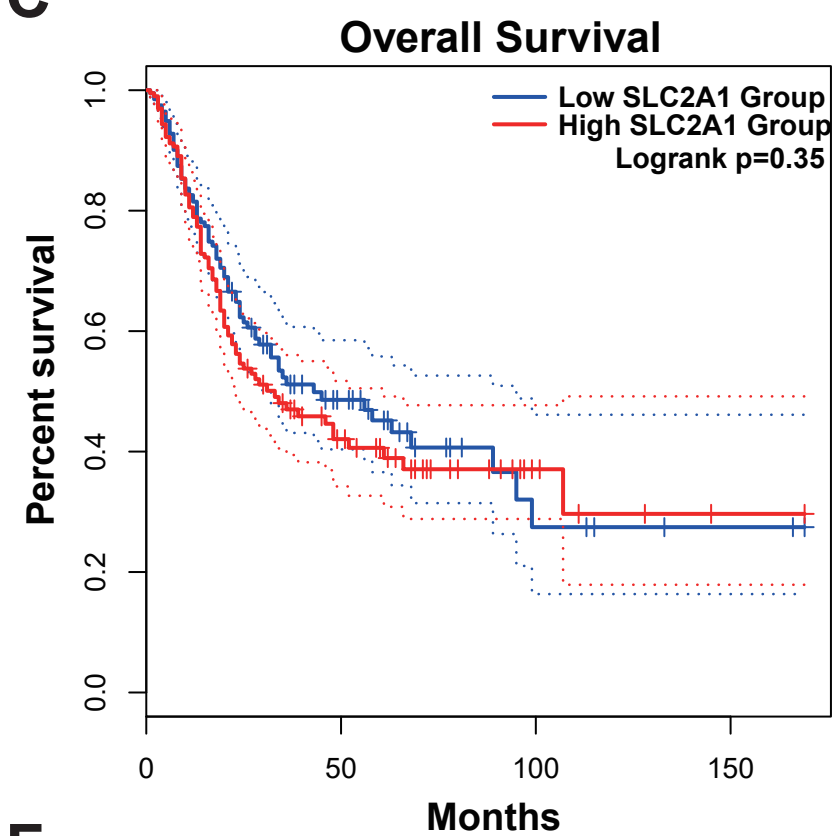**D**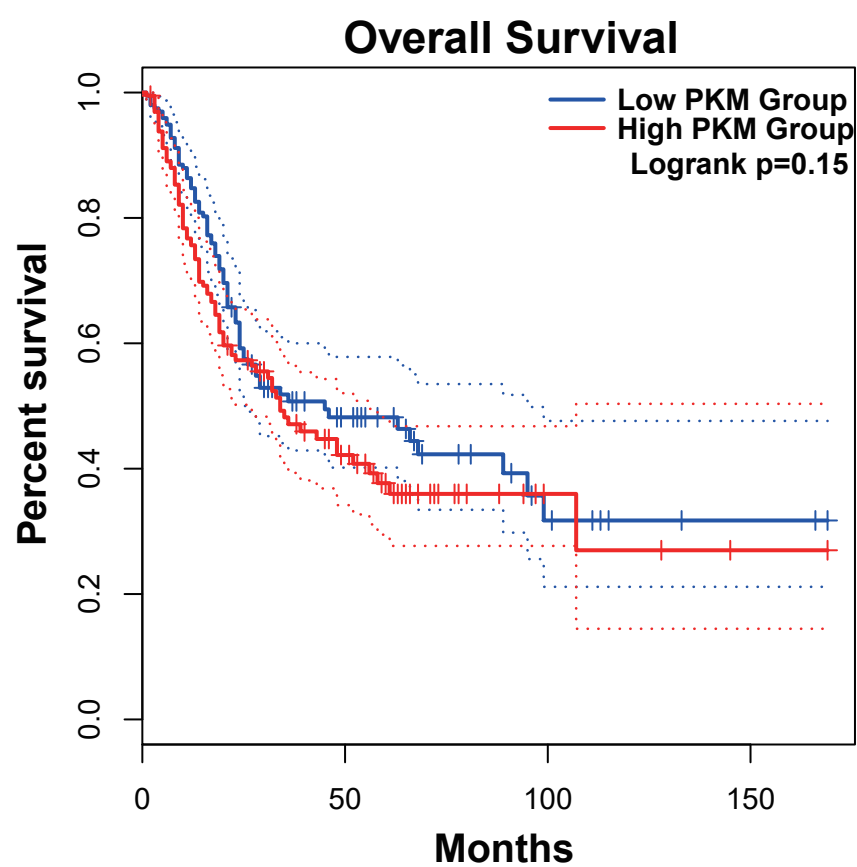**E**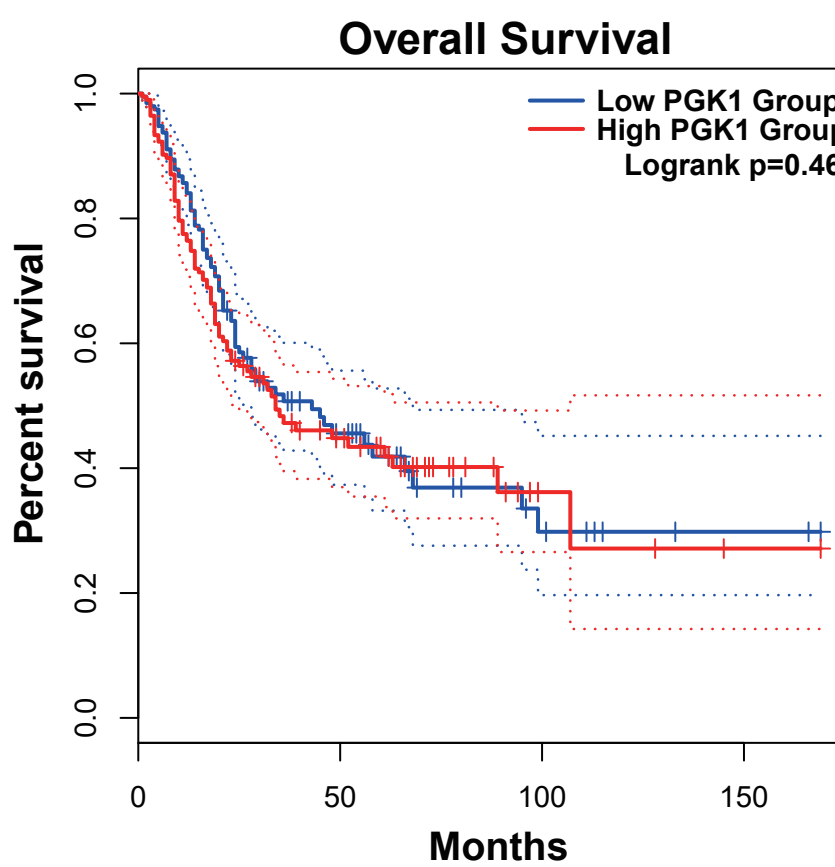**F**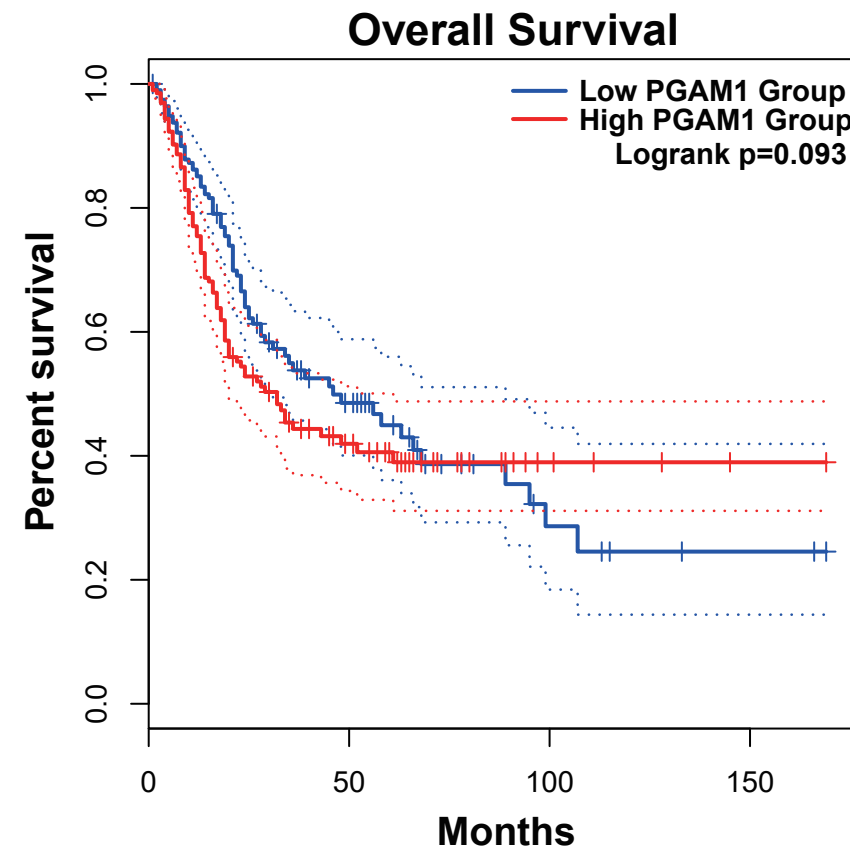**G**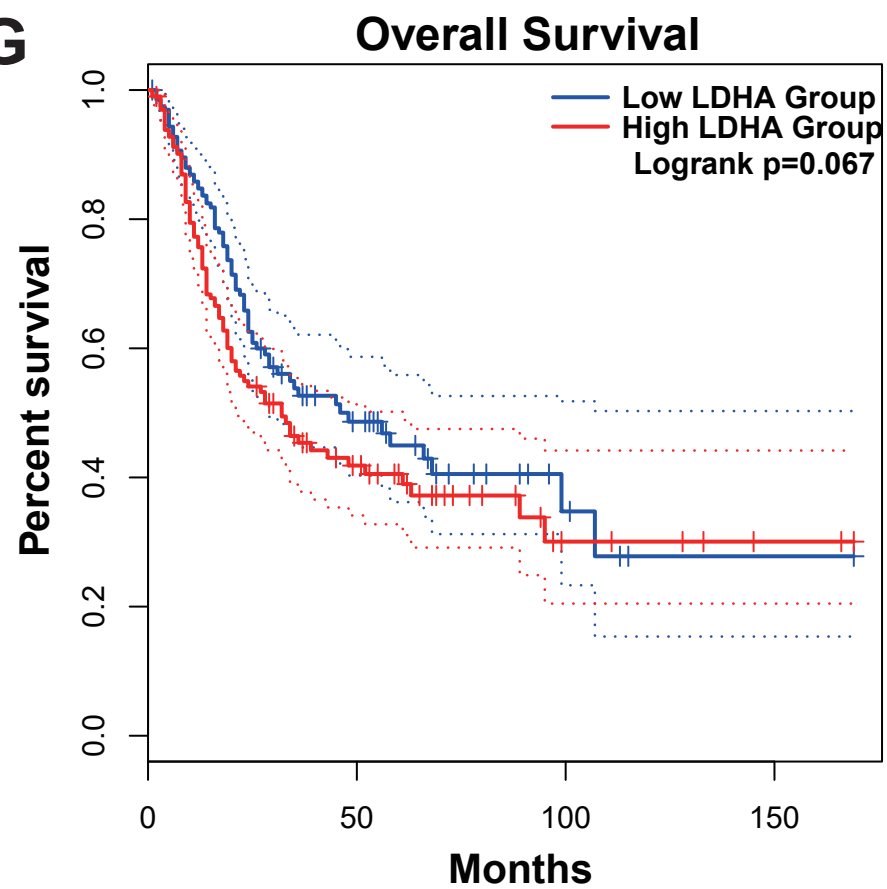**H**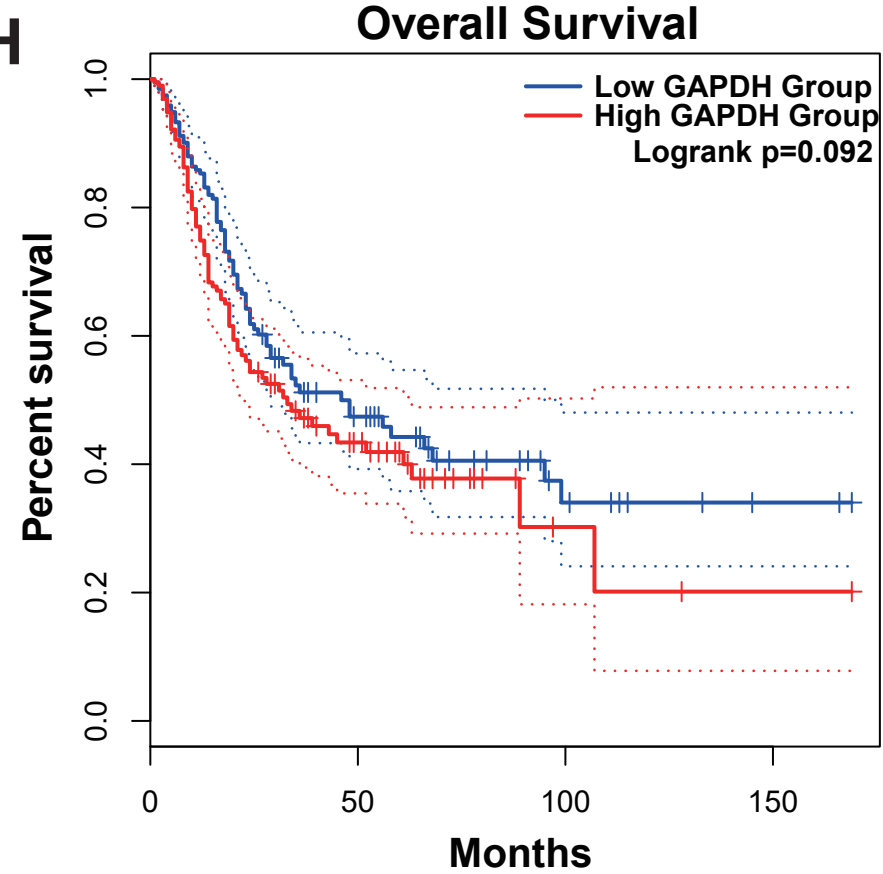

Supplement: Supplementary Materials — Supplemental Figure 1: classification of 20 methylation regulators. Supplemental Figure 2: validation of 3 hub m6A regulators. (a) Overall survival rate of FTO in BLCA. (b) Overall survival rate of IGF2BP3 in BLCA. (c) Overall survival rate of YTHDC1 in BLCA. Supplemental Figure 3: correlation of 3 m6A hub methylation regulators with clinicopathological characteristics. (a) Distribution of FTO expression in grade, stage, T, and fustat. P=0.0036, 0.00078, 0.0071, and 0.011, respectively, by Kruskal–Wallis rank-sum t. (b) Distribution of IGF2BP3 expression in grade, stage, T, and fustat. P=2.7e − 07, 0.0036, 0.0012, and 0.0017, respectively, by Kruskal–Wallis rank-sum t. (c) Distribution of YTHDC1 expression in age and fustat. P=0.01 and 0.078, respectively, by Kruskal–Wallis rank-sum t. Supplemental Figure 4: consensus clustering analysis based on 3 m6A hub methylation regulators vs. 19 m6A methylation regulators. (a) Consensus clustering distribution function (CDF) for bladder cancer. (b) Relative changes in the area under the CDF curve for bladder cancer. (c) Principal component analysis (PCA) for total RNA expression pattern. Subgroups are marked with blue and red. (d) Consensus clustering matrix for BLCA based on 19 m6A methylation regulators. (e) The Kaplan–Meier OS curves for the two subgroups. P=0.155 (cluster 1 vs. cluster 2). (f) Principal component analysis (PCA) for total RNA expression pattern. Subgroups are marked with blue and red. Supplemental Figure 5: survival analysis of 10 hub genes in cluster 1. (a–h) Overall survival rates of 8 hub genes in cluster 1. (a) TPL1, P=0.062. (b) SOD2, P=0.57. (c) SLC2A1, P=0.35. (d) PKM, P=0.15. (e) PGK1, P=0.46. (f) PGAM1, P=0.093. (g) LDHA, P=0.067. (h) GAPDH, P=0.092. Supplemental Figure 6: 3 m6A hub methylation regulators are significantly related to immune infiltration. (a–c) The relationship of FTO, IGF2BP3, and YTHDC1 with macrophage 2. (d–f) The relationship of FTO, IGF2BP3, and YTHDC1 with TFH. [file 8581805.f1.zip › 8581805.f1/Supplemental5.pdf]

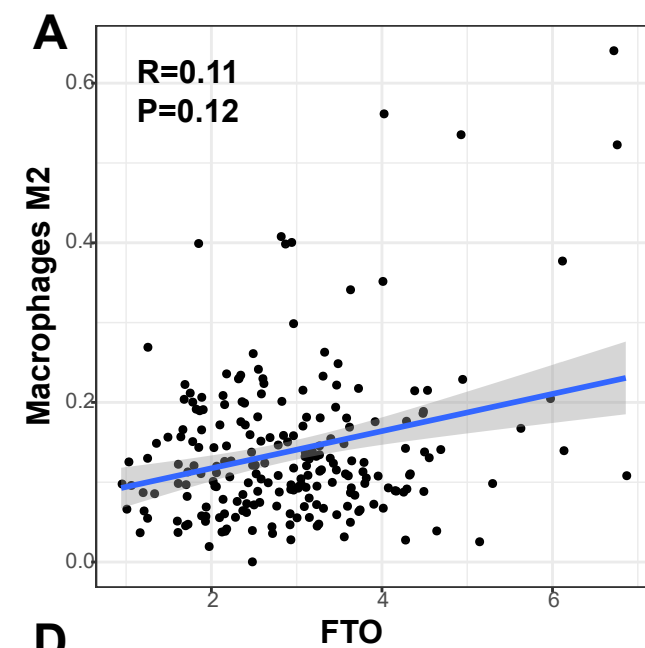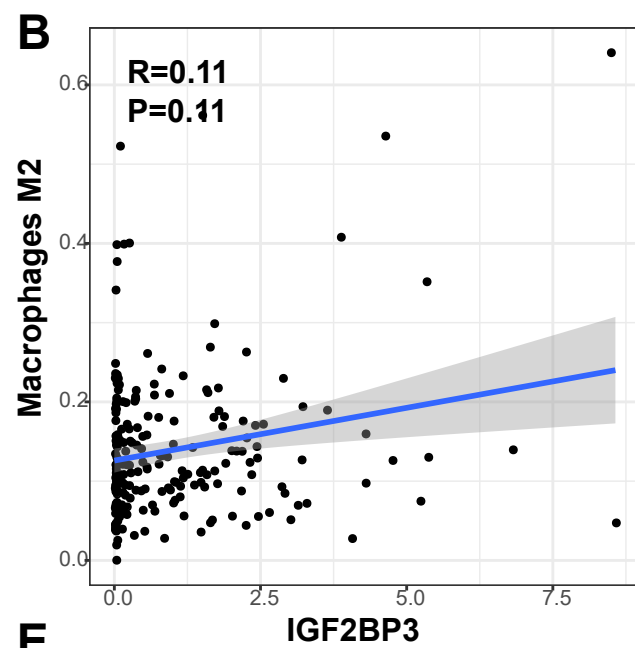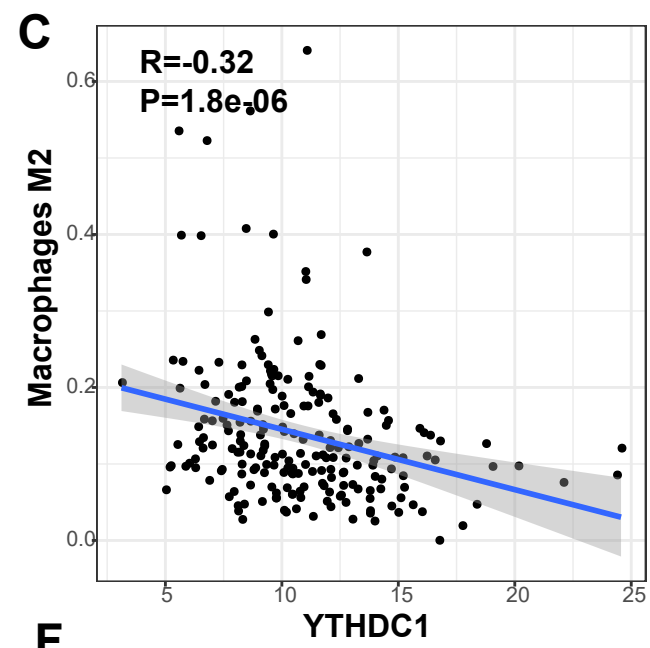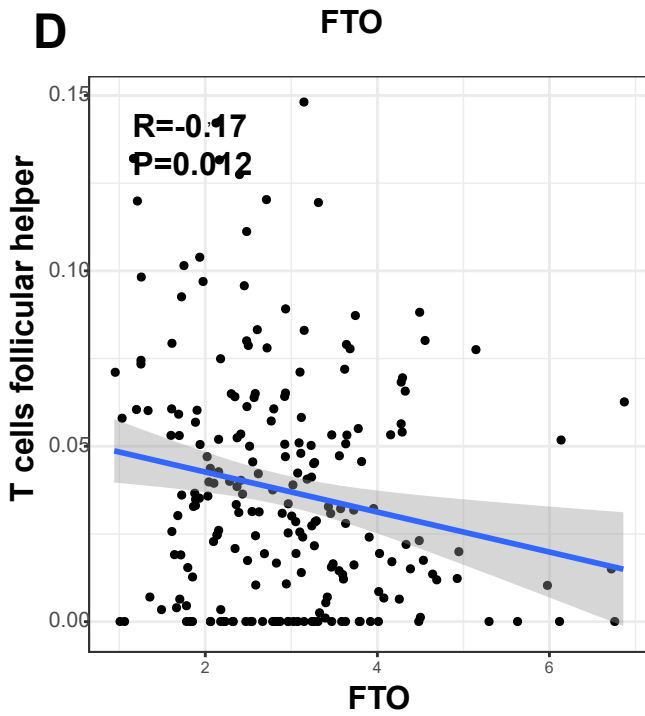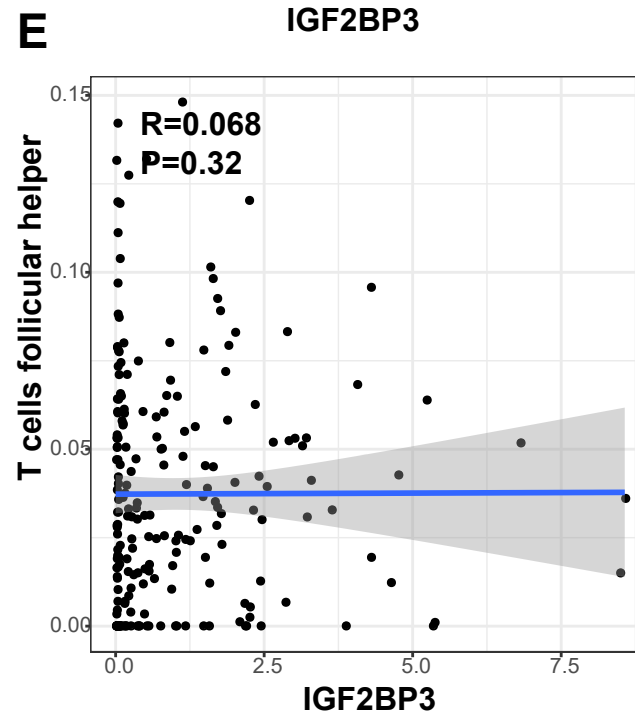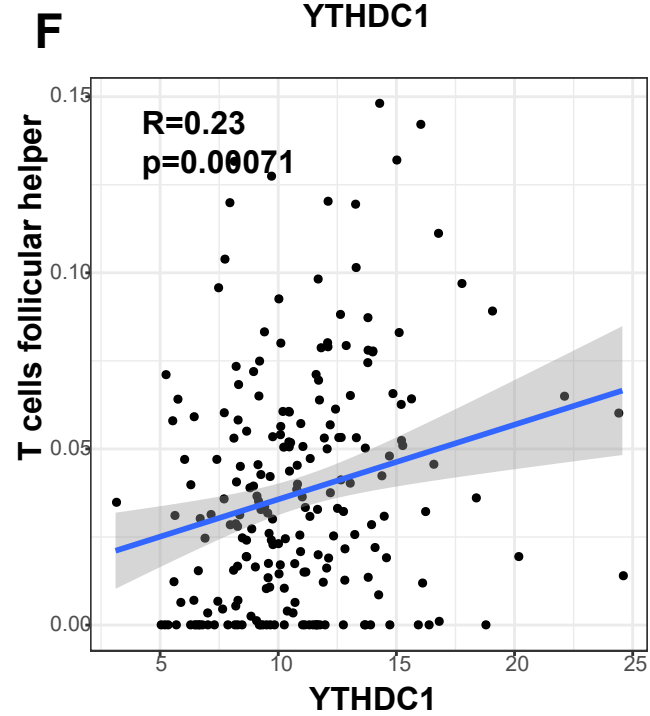

Supplement: Supplementary Materials — Supplemental Figure 1: classification of 20 methylation regulators. Supplemental Figure 2: validation of 3 hub m6A regulators. (a) Overall survival rate of FTO in BLCA. (b) Overall survival rate of IGF2BP3 in BLCA. (c) Overall survival rate of YTHDC1 in BLCA. Supplemental Figure 3: correlation of 3 m6A hub methylation regulators with clinicopathological characteristics. (a) Distribution of FTO expression in grade, stage, T, and fustat. P=0.0036, 0.00078, 0.0071, and 0.011, respectively, by Kruskal–Wallis rank-sum t. (b) Distribution of IGF2BP3 expression in grade, stage, T, and fustat. P=2.7e − 07, 0.0036, 0.0012, and 0.0017, respectively, by Kruskal–Wallis rank-sum t. (c) Distribution of YTHDC1 expression in age and fustat. P=0.01 and 0.078, respectively, by Kruskal–Wallis rank-sum t. Supplemental Figure 4: consensus clustering analysis based on 3 m6A hub methylation regulators vs. 19 m6A methylation regulators. (a) Consensus clustering distribution function (CDF) for bladder cancer. (b) Relative changes in the area under the CDF curve for bladder cancer. (c) Principal component analysis (PCA) for total RNA expression pattern. Subgroups are marked with blue and red. (d) Consensus clustering matrix for BLCA based on 19 m6A methylation regulators. (e) The Kaplan–Meier OS curves for the two subgroups. P=0.155 (cluster 1 vs. cluster 2). (f) Principal component analysis (PCA) for total RNA expression pattern. Subgroups are marked with blue and red. Supplemental Figure 5: survival analysis of 10 hub genes in cluster 1. (a–h) Overall survival rates of 8 hub genes in cluster 1. (a) TPL1, P=0.062. (b) SOD2, P=0.57. (c) SLC2A1, P=0.35. (d) PKM, P=0.15. (e) PGK1, P=0.46. (f) PGAM1, P=0.093. (g) LDHA, P=0.067. (h) GAPDH, P=0.092. Supplemental Figure 6: 3 m6A hub methylation regulators are significantly related to immune infiltration. (a–c) The relationship of FTO, IGF2BP3, and YTHDC1 with macrophage 2. (d–f) The relationship of FTO, IGF2BP3, and YTHDC1 with TFH. [file 8581805.f1.zip › 8581805.f1/Supplemental6.pdf]
